# Early life exposure to economic shocks and association with childhood malnutrition: a pooled analysis of 230 nationwide surveys from 68 low-income and middle-income countries

**DOI:** 10.1016/S2214-109X(25)00153-6

**Published:** 2025-06-27

**Authors:** Natanael J Silva, Enny S Paixão, Nicolai Brachowicz, Gonzalo Barreix, Elisa Landin-Basterra, Felipe A Rubio, Delia Boccia, Rita C Ribeiro-Silva, Mauricio L Barreto, Aliya Naheed, Ivalda Macicame, Denise Naniche, Davide Rasella

**Affiliations:** a Barcelona Institute for Global Health (ISGlobal), Barcelona, Spain; b Facultat de Medicina i Ciències de la Salut, Universitat de Barcelona, Barcelona, Spain; c Faculty of Epidemiology and Population Health, London School of Hygiene & Tropical Medicine, London, UK; d Faculty of Population and Health Policy, London School of Hygiene & Tropical Medicine, London, UK; e Instituto de Saúde Coletiva, Universidade Federal da Bahia, Salvador, Brazil; f Escola de Nutrição, Universidade Federal da Bahia, Salvador, Brazil; g Centro de Integração de Dados e Conhecimentos para Saúde, Fundação Oswaldo Cruz, Salvador, Brazil; h International Centre for Diarrhoeal Disease Research, Dhaka, Bangladesh; i Instituto Nacional de Saúde, Maputo, Mozambique; j Institución Catalana de Investigación y Estudios Avanzados (ICREA), Barcelona, Spain

## Abstract

**Background:**

The relationship between economic growth and nutrition is not yet fully understood in the context of the new nutrition reality where most low-income and middle-income countries (LMICs) face an increasing double burden of malnutrition. We aimed to investigate the association between early life exposure to economic shocks and multiple forms of childhood malnutrition in LMICs.

**Methods:**

We pooled cross-sectional data on children younger than 5 years from the Demographic and Health Surveys from 1990 to 2022 and longitudinal income data from the World Inequality Database. An economic shock was defined as any negative income growth and was tested at intensity levels of less than –1%, –5%, and –10%. Malnutrition outcomes variables comprised stunting, wasting, overweight, obesity, concurrent wasting and stunting (WaSt), and double burden of malnutrition (child is both stunted and overweight). Adjusted associations of economic shocks, at different critical windows (year of the interview, birth, first 1000 days of life), with malnutrition outcomes were estimated by multivariable Poisson regression models with robust errors. The associations were interpreted using prevalence ratios (PRs) and 95% CIs.

**Findings:**

A total of 1 643 898 children across 230 surveys in 68 LMICs were included in this study. Negative income shocks in the year of interview were associated with a 5·4% (PR 1·054 [95% CI 1·029–1·080]) increase in the prevalence of wasting and a 12·7% (1·127 [1·079–1·176]) increase in severe wasting. A dose–response association according to the intensity levels of income shock in the year of birth was found for stunting (–1%: PR 1·019 [95% CI 1·011–1·027]; –5%: 1·033 [1·025–1·042]; and –10%: 1·061 [1·051–1·072]) and severe stunting (–1%: 1·041 [1·026–1·055]; –5%: 1·059 [1·044–1·074]; and –10%: 1·099 [1·081–1·118]). In children aged 2–4 years, income shocks during the first 1000 days of life strongly increased the prevalence of double burden of malnutrition by 30·3% (PR 1·303 [95% CI 1·221–1·391]), obesity by 14·3% (1·143 [1·046–1·249]), and overweight by 13·8% (1·138 [1·090–1·188]). We also found a strong dose–response association between the intensity of income shock during the first 1000 days and double burden of malnutrition (–1%: PR 1·216 [95% CI 1·141–1·295]; –5%: 1·299 [1·192–1·416]; –10%: 1·350 [1·185–1·537]).

**Interpretation:**

Exposure to negative income shocks can significantly increase the risk of various forms of malnutrition during childhood, with critical windows of vulnerability that vary based on the timing of economic instability and the specific type of malnutrition. Policymakers and public health practitioners must recognise these critical periods and develop targeted interventions to safeguard maternal and child nutrition, particularly during times of economic crises.

**Funding:**

Ministry of Science, Innovation and Universities of Spain; Spanish State Research Agency; Generalitat de Catalunya through the CERCA Program; and Wellcome Trust.

## Introduction

The world is currently facing a polycrisis characterised by a confluence of economic, social, and environmental challenges.^1^, ^2^ These crises have profound impacts on individuals and disproportionately affect their nutrition, health, and wellbeing, particularly children and women in low-income and middle-income countries (LMICs).^3^ Increases in poverty, food insecurity, and limited access to essential goods, such as food, health, water and sanitation, education, and social protection, have exacerbated the risk of childhood malnutrition and stalled progress toward achieving the 2025 World Health Assembly Global Nutrition targets and the 2030 Sustainable Development Goals, including ending hunger and malnutrition in all its forms.^4^

A recent report states that one-in-four children worldwide, equivalent to 181 million children younger than 5 years, is living in severe child food poverty.^5^ In 2022, 148 million children were stunted, 45 million were wasted, and another 37 million were living with overweight or obesity.^6^ Two recent studies have examined the effect of economic shocks and food inflation on child undernutrition outcomes in LMICs.^7^, ^8^ One study predicted a 14·4–17·8% increase in child wasting prevalence following a 10% annual decline in national income per capita,^7^ and the other study estimated that a 5% increase in the real price of food raises the risk of wasting by 9% and severe wasting by 14%.^8^

**Research in context**

**Evidence before this study**

We searched PubMed for studies published in any language from database inception to Sept 16, 2024, using the search terms: “(“Economic Shock*” OR “Income Shock*” OR “Economic Cris*” OR “Economic Downturn*” OR “Economic Recession”[MeSH Terms] OR “Financial Stress”[MeSH Terms] OR “Fiscal Cris*” OR “Financial Cris*” OR “Economic Insecurity” OR “Economic Instability” OR “Macroeconomic Shock*”) AND (“Malnutrition” OR “Stunting” OR “Wasting” OR “Overweight” OR “Obesity” OR “Undernutrition” OR “Overnutrition” OR “Double Burden of Malnutrition” OR “Nutrition Disorders”[MeSH Terms] OR “Nutritional Status”[MeSH Terms] OR “Body Height”[MeSH Terms] OR “Body Weight”[MeSH Terms])”. We identified 354 articles, of which only ten provided evidence on the association between economic crises or shocks and child growth outcomes in low-income and middle-income countries (LMICs). Most studies focused on specific countries, including Indonesia (n=3), China (n=2), Ethiopia and Nigeria (n=1), Cameroon (n=1), Congo (Brazzaville; n=1), and Brazil (n=1). To date, only one multi-country study has examined the relationship between country-level economic shocks and child wasting outcomes, estimating a 14·4–17·8% increase in wasting prevalence following a 10% annual decline in national income per capita.

**Added value of this study**

Our study examined the association between early life exposure to economic shocks and multiple forms of childhood malnutrition, using pooled data from 230 nationwide surveys across 68 LMICs, totalling more than 1·6 million children. We demonstrate that exposure to negative income shocks can significantly increase the risk of various forms of malnutrition during childhood, with dose–response associations and critical windows of vulnerability that vary based on the timing of economic instability and the specific type of malnutrition. Our research makes a novel contribution to the existing literature by employing a disaggregated measure of national income shocks that accounts for the economic heterogeneity within the population of each country, and by studying for the first time critical windows, multiple malnutrition outcomes, and the double burden of malnutrition.

**Implications of all the available evidence**

Policymakers and public health practitioners must recognise the critical windows of development, such as pregnancy and the first 1000 days of life, and develop targeted interventions to safeguard maternal and child nutrition, particularly in the current times of polycrisis. Furthermore, tailored strategies are needed to address all forms of malnutrition across different socioeconomic groups, ensuring that all children have the opportunity to achieve optimal growth and development.

Despite extensive literature on economic growth and undernutrition,^7^, ^9^, ^10^, ^11^ the relationship between economic hardship and other nutritional outcomes remains not fully understood in the context of the new nutrition reality. Most LMICs now face an increasing double burden of malnutrition, characterised by the coexistence of undernutrition alongside overweight, obesity, or diet-related non-communicable diseases.^12^ This dual burden poses unique challenges and calls for a comprehensive life course approach to better understand how economic shocks and poverty influence various forms of malnutrition, providing insights into optimal timing for interventions and primary prevention strategies.^13^, ^14^

In this study, we aimed to investigate the association between the early life exposure to economic shocks and multiple forms of malnutrition during childhood in LMICs, and to identify critical windows and population subgroups of vulnerability to target for future preventive efforts.

## Methods

### Data and study design

We pooled cross-sectional individual data from the Demographic and Health Surveys (DHS) and national-level longitudinal economic data from the World Inequality Database (WID).

DHS are nationally representative household surveys that provide accurate data on demographics and health in more than 90 countries worldwide. From the DHS recode files of children, we obtained information about the pregnancy and postnatal care and health of children younger than 5 years, as well the data for the mother and household of each of these children. We only included surveys conducted from 1990 to 2022 in LMICs (World Bank classification in 2022), with complete anthropometric and wealth index information.

The WID is an extensive and open database on the world distribution of income and wealth, both between and within countries. This database is primarily maintained by the World Inequality Lab at the Paris School of Economics. We obtained annual national average estimates of pretax income for each decile of the country's income distribution from the WID to estimate economic shocks.

### Outcome variables

Using height (cm) and weight (kg) measurements of all children aged 0–59 months from DHS, we calculated height-for-age Z scores (HAZ) and weight-for-height Z scores (WHZ) based on WHO Child Growth Standards curves using the WHO macro package igrowup available for Stata.^15^ More details about the calculation of anthropometric Z scores are provided in the appendix (p 2). We excluded observations with missing and biologically implausible values for height and weight. Biologically implausible values were considered as HAZ less than –6 or more than 6 and WHZ less than –6 or more than 5.^16^

Malnutrition outcomes were classified in accordance with WHO cutoffs^15^ and comprised the following: stunting (HAZ less than –2), severe stunting (HAZ less than –3), wasting (WHZ less than –2), severe wasting (WHZ less than –3), overweight (WHZ >2), and obesity (WHZ >3). In addition, we analysed the coexistence of different forms of malnutrition in the same child: concurrent wasting and stunting (WaSt; WHZ less than –2 and HAZ less than –2); and stunting and overweight (HAZ less than –2 and WHZ >2), an individual-level manifestation of the double burden of malnutrition.

### Main exposure variables

Pretax income estimates for each decile of the country's income distribution were annually obtained from the WID and then matched with DHS individual data using information about country of residence, year (interview, birth, and all years within the first 1000 days), and wealth index decile (appendix pp 3–4). We matched these two datasets based on the proxy assumption of socioeconomic status position that individuals in a certain wealth decile might be ranked in the same income decile.^17^ Similar approaches have already been applied in other studies using DHS data.^18^, ^19^

An economic shock event was defined as a binary variable indicating any instance of negative income growth within a specific wealth decile, irrespective of income changes in other deciles. On this basis, we calculated the annual percentage growth rates of pretax income values in US$ purchasing power parity (PPP), which were previously transformed from national currency units using the country–year US$ PPP conversion factors provided by the WID.^20^ To test different intensity levels of economic shocks, we also analysed negative income growth less than –1%, –5%, and –10%.

The temporal effects of economic shocks can vary across forms of malnutrition. Child wasting is particularly sensitive to short-term shocks such as declines in diet quality or quantity, infections, or other illnesses.^21^ In contrast, child stunting captures the cumulative or long-term effects of poor nutrition, including repeated episodes of wasting, during the critical window of the first 1000 days of life (from conception to 2 years).^21^ Although the literature extensively explores these two forms of undernutrition, understanding the dynamics of other forms of malnutrition and their coexistence (eg, double burden of malnutrition) in the context of economic shocks remains poor. Therefore, we explored different exposure windows: income shocks in the same year as the interview, in the year of birth, and across all years within the first 1000 days of life. Because only children 2 years or older were completely exposed to the critical period of the first 1000 days, we restricted the analyses for this time window to children aged 2–4 years only. All children younger than 5 years were included in the analyses for the year of the interview and the year of birth.

### Covariates

A set of relevant confounding variables at the individual and household level was selected based on data availability and completeness, and based on the literature of economic crises and child nutrition. The covariates in the analysis comprised the following: rural versus urban place of residence, unimproved versus improved toilet facility, wealth index quintiles (q1 [poorest], q2, q3, q4, and q5 [richest]), maternal age at birth (years), maternal education equal or less than 5 years versus more than 5 years, maternal parity equal to or greater than three live births versus less than three births, female versus male child, child's age (months), and whether the child was born at a medical facility versus others.

An unimproved toilet facility was defined as a facility that flushed to a known location but not to a sewer system, septic tank, or pit latrine.^22^ Wealth index quintiles were based on a household wealth score provided by the DHS, which considers the ownership of assets, building materials, electricity availability, and types of water access and sanitation facilities.^22^ Maternal education was defined based on the sample's median number of schooling years and parity was defined based on the sample's median number of live births.

### Statistical analysis

The associations between economic shocks at different exposure time windows (interview, birth, first 1000 days) and the binary outcomes of child malnutrition were estimated using multivariable Poisson regression models with robust standard errors. All models were based on complete case analyses, weighted, and adjusted for confounding control as well as country-fixed and time-fixed effects.

The following equation describes our model:

*Log(ChM*_ict_*)=*β_0_ + β_1_*ES*_ict_ + Σβ_n_*X*_nict_ + β_c_*C*_c_ + β_t_*T*_t_ + ε_ict_

where *ChM* is the malnutrition outcome for the child (*i*), in country (*c*), during interview year (*t*), *ES* is the economic shock at a specific timepoint (interview, birth, or the first 1000 days), Σ*X*_n_ is the vector of *n* confounding variables from DHS, *C* is the country-fixed effects, *T* is the year-fixed effects, and ε is the error term.

Although using a Poisson distribution to model a binary outcome might seem counterintuitive, this robust Poisson method is a semiparametric model that does not assume a specific distribution for the outcome. Instead, this model only assumes a log-linear relation between the risk or prevalence of the outcome and the explanatory variables. Methodological papers have shown that this method provides a good approximation to the more conventional binomial distribution, particularly when the outcome is not rare (eg, prevalence >10%).^23^ The associations were interpreted using prevalence ratios (PRs) with their 95% CIs. A PR >1 indicates a higher prevalence of the outcome in the exposed group compared with the unexposed group, whereas a PR <1 suggests a lower prevalence.

Similar to previous multi-country studies using DHS data, survey-specific weights were rescaled and applied to the models to enable cross-country and cross-survey comparisons.^7^, ^8^ Moreover, all models were adjusted for the aforementioned set of confounding variables from DHS, as well as for country and year dummies to account for fixed effects. On the one hand, country-fixed effects control for unobserved, constant differences across countries (eg, geographical, cultural, and socioeconomic characteristics). On the other hand, time-fixed effects capture changes over time that affect all countries similarly (eg, global economic and social trends). By including these fixed effects, we reduced the bias from omitted variables that could confound the association between economic shocks and child malnutrition.

We hypothesise that the relationship between income shocks and child malnutrition might be different across socioeconomic groups. Therefore, we also estimated models separately for each wealth quintile. To statically justify this stratified analysis and support our hypothesis, we further estimated models adding an interaction term between income shock and wealth quintiles and assessed its significance using both a Wald test and a likelihood-ratio test.

Sensitivity and triangulation analyses were conducted to assess the robustness of the results. First, we ran bivariate models to examine the association between the main exposure and outcome variables. Second, we performed subset analyses, including only surveys conducted from 2000 to 2019, to account for improved data since 2000 and to eliminate potential residual effects of the COVID-19 pandemic. Third, we adjusted the models for two country-level variables, using the same exposure windows as for economic shocks, to account for the potential impact of conflict and fragile situations: a national state of emergency due to a natural disaster and a national state of emergency due to armed conflict or war (domestic or international). These variables were extracted from the Varieties of Democracy database and represent the mean dichotomous assessment of multiple experts on the existence of an emergency state in each country-year.^24^ Fourth, to assess the effect of clustering from multiple children within the same household, we re-ran the main models using only the eldest child per household. Finally, we conducted a triangulation analysis by using linear models with HAZ and WHZ as continuous dependent variables, applying the same specifications as in the main analysis.

We did the data processing and analyses using R (version 4.3.0) and Stata (version 14.0).

### Role of the funding source

The funders of the study had no role in study design, data collection, data analysis, data interpretation, or writing of the report.

## Results

Our final study sample included a total of 1 643 898 children younger than 5 years, assessed across 230 surveys in 68 LMICs between 1990 and 2022 (appendix p 5). Most of the surveys were from countries located in west and central Africa, eastern and southern Africa, Latin America, south Asia, and eastern Europe and central Asia (appendix pp 6–9). Most of the households in the sample were situated in rural areas and had improved toilet facilities (table 1). On average, mothers were 26 years old at the time of birth, and approximately half of them had less than 5 years of education and had given birth to three or more live newborns. The children were, on average, 28 months' old at the time of interview and were mostly born in medical facilities.

**Table 1:** Descriptive statistics of the study variables

		**n (%)**
**Total**		
Number of children		1 643 898
Number of mothers		1 268 432
Number of households		1 186 948
Number of surveys		230
Number of countries		68
**Global region**		
South Asia		566 341 (34·5%)
West and central Africa		368 098 (22·4%)
Eastern and southern Africa		302 559 (18·4%)
Latin America and Caribbean		228 793 (13·9%)
Middle East and north Africa		97 254 (5·9%)
Eastern Europe and central Asia		46 553 (2·8%)
East Asia and Pacific		34 300 (2·1%)
**Country income level**		
Low income		294 453 (17·9%)
Lower-middle income		1 130 499 (68·8%)
Upper-middle income		218 946 (13·3%)
**Malnutrition outcomes**		
Stunting (HAZ less than −2)		583 421 (35·5%)
Severe stunting (HAZ less than −3)		254 139 (15·5%)
Wasting (WHZ less than −2)		182 155 (11·1%)
Severe wasting (WHZ less than −3)		66 595 (4·1%)
Overweight (WHZ >2)		80 470 (4·9%)
Obesity (WHZ >3)		24 675 (1·5%)
WaSt (WHZ less than −2 and HAZ less than −2)		58 704 (3·6%)
DBM (HAZ less than −2 and WHZ >2)		39 008 (2·4%)
**Economic shocks**		
Year of the interview		
	Any negative income shock	827 140 (50·3%)
	Negative income shock less than −1%	693 526 (42·2%)
	Negative income shock less than −5%	443 740 (27·0%)
	Negative income shock less than −10%	181 136 (11·0%)
Year of birth		
	Any negative income shock	1 013 061 (61·6%)
	Negative income shock less than −1%	905 811 (55·1%)
	Negative income shock less than −5%	463 177 (28·2%)
	Negative income shock less than −10%	239 500 (14·6%)
All years of the first 1000 days*		
	Any negative income shock	346 532 (36·6%)
	Negative income shock less than −1%	256 005 (27·0%)
	Negative income shock less than −5%	85 258 (9·0%)
	Negative income shock less than −10%	43 046 (4·6%)
**Household characteristics**		
Rural place of residence		1 120 860 (68·2%)
Unimproved toilet facility		676 877 (41·2%)
Wealth quintile (poorest)		332 647 (20·2%)
**Maternal characteristics**		
Maternal education ≤5 years		879 377 (53·5%)
Maternal parity ≥3 births		911 179 (55·4%)
Maternal age at birth in years		26 (6·3)
**Child characteristics**		
Child's age in months		28 (17·0)
Female child		808 169 (49·2%)
Child born at medical facility		1 022 492 (62·2%)

Data are n (%), n, or mean (SD). DBM=double burden of nutrition. HAZ=height-for-age Z score. WaSt=concurrent wasting and stunting. WHZ=weight-for-height Z score.

* Analyses for the critical period of the first 1000 days of life were restricted to children aged 2–4 years only (n=946 804).

Overall, the prevalence of stunting was 35·5% and severe stunting was 15·5% (table 1). The prevalence of wasting was 11·1% and severe wasting was 4·1%, whereas the prevalence of overweight was 4·9% and obesity was 1·5%. The prevalence of WaSt was 3·6%, and double burden of malnutrition was 2·4%. Based on the most recent survey of each country, the countries with the highest prevalence of undernutrition (stunting, wasting, and WaSt) were located in south Asia, east Asia and Pacific, eastern and southern Africa, and west and central Africa (appendix pp 10–11). The prevalence of overweight and double burden of malnutrition were higher in the Middle East as well as eastern Europe and central Asia.

Exposure to economic shocks was most frequent in the year of birth, affecting 61·6% of children, followed by the year of the interview, in which 50·3% were exposed to a negative income shock (table 1). Among children 2 years or older, 36·6% were exposed to a negative income shock in all years of their first 1000 days of life. When the average income growth dropped by more than 1% and 10%, the exposure rates were 42·2% and 11·0% in the year of the interview, 55·1% and 14·6% in the year of birth, and 36·6% and 4·6% throughout the first 1000 days of life, respectively. Regardless of the exposure time, the countries most affected by economic shocks were situated in eastern and southern Africa, Middle East and north Africa, central Asia, and Latin America (appendix pp 12–13).

Adjusted analyses show that negative income shocks in the year of the interview were associated with a 5·4% (PR 1·054 [95% CI 1·029–1·080]) increase in the prevalence of wasting and a 12·7% (1·127 [1·079–1·176]) increase of severe wasting (table 2). Similarly, income shocks in the year of the interview increased the prevalence of overweight by 3·6% (PR 1·036 [95% CI 1·007–1·067]) and obesity by 9·3% (1·093 [1·029–1·161]). To a lesser extent, income shocks were also positively associated with severe stunting (PR 1·020 [95% CI 1·003–1·037]). Full models are shown in the appendix (p 14). Negative income shocks less than –1%, –5%, and –10% were significantly associated with several outcomes, particularly wasting and severe wasting, but no dose–response association was observed.

**Table 2:** Adjusted association between exposure to income shocks at different time windows and child malnutrition outcomes by intensity of income shock

	**Stunting (HAZ less than −2)**		**Severe stunting (HAZ less than −3)**		**Wasting (WHZ less than −2)**		**Severe wasting (WHZ less than −3)**		**Overweight (WHZ >2)**		**Obesity (WHZ >3)**		**WaSt (WHZ less than −2 and HAZ less than −2)**		**DBM (HAZ less than −2 and WHZ >2)**	
	PR (95% CI)	p value	PR (95% CI)	p value	PR (95% CI)	p value	PR (95% CI)	p value	PR (95% CI)	p value	PR (95% CI)	p value	PR (95% CI)	p value	PR (95% CI)	p value
Year of the interview*																
Any negative income shock	0·995 (0·986–1·005)	0·334	1·020 (1·003–1·037)	0·021	1·054 (1·029–1·080)	<0·001	1·127 (1·079–1·176)	<0·001	1·036 (1·007–1·067)	0·016	1·093 (1·029–1·161)	0·004	1·027 (0·987–1·070)	0·189	1·046 (0·997–1·097)	0·067
Negative income shock less than −1%	1·007 (0·998– 1·017)	0·113	1·036 (1·019–1·052)	<0·001	1·047 (1·022–1·072)	<0·001	1·100 (1·052–1·149)	<0·001	1·025 (0·997–1·053)	0·081	1·039 (0·985–1·096)	0·159	1·037 (0·996–1·080)	0·077	1·042 (0·999–1·088)	0·057
Negative income shock less than −5%	1·029 (1·019–1·039)	<0·001	1·072 (1·054–1·092)	<0·001	1·095 (1·068–1·122)	<0·001	1·198 (1·146–1·252)	<0·001	1·017 (0·986–1·049)	0·282	1·019 (0·960–1·082)	0·533	1·059 (1·014–1·105)	0·010	1·101 (1·051–1·155)	<0·001
Negative income shock less than −10%	1·010 (0·997–1·024)	0·121	1·006 (0·983–1·029)	0·630	1·060 (1·024–1·098)	0·001	1·079 (1·014–1·149)	0·017	0·954 (0·914–0·995)	0·030	0·932 (0·857–1·014)	0·103	1·086 (1·021–1·155)	0·009	1·002 (0·938–1·071)	0·951
Year of birth*																
Any negative income shock	1·027 (1·019–1·036)	<0·001	1·061 (1·045–1·076)	<0·001	1·035 (1·014–1·056)	0·001	1·033 (0·995–1·072)	0·087	1·039 (1·012–1·068)	0·005	1·051 (0·998–1·108)	0·059	1·053 (1·017–1·091)	0·003	1·049 (1·006–1·093)	0·025
Negative income shock less than −1%	1·019 (1·011–1·027)	<0·001	1·041 (1·026–1·055)	<0·001	1·027 (1·007–1·048)	0·007	1·017 (0·981–1·055)	0·348	1·035 (1·010–1·062)	0·007	1·039 (0·989–1·092)	0·124	1·054 (1·019–1·091)	0·002	1·037 (0·997–1·078)	0·067
Negative income shock less than −5%	1·033 (1·025–1·042)	<0·001	1·059 (1·044–1·074)	<0·001	1·098 (1·075–1·121)	<0·001	1·143 (1·100–1·186)	<0·001	0·997 (0·972–1·023)	0·827	1·005 (0·956–1·056)	0·851	1·115 (1·075–1·157)	<0·001	1·041 (1·001–1·082)	0·043
Negative income shock less than −10%	1·061 (1·051–1·072)	<0·001	1·099 (1·081–1·118)	<0·001	1·055 (1·027–1·084)	<0·001	1·078 (1·028–1·130)	0·002	0·994 (0·962–1·027)	0·732	0·949 (0·889–1·013)	0·117	1·106 (1·056–1·159)	<0·001	1·072 (1·022–1·124)	0·004
**All years of the first 1000 days**†																
Any negative income shock	1·023 (1·012–1·033)	<0·001	1·052 (1·033–1·072)	<0·001	1·029 (0·990–1·068)	0·146	1·018 (0·952–1·089)	0·602	1·138 (1·090–1·188)	<0·001	1·143 (1·046–1·249)	0·003	1·019 (0·959–1·083)	0·549	1·303 (1·221–1·391)	<0·001
Negative income shock less than −1%	1·006 (0·995–1·017)	0·272	1·029 (1·010–1·049)	0·002	1·046 (1·006–1·088)	0·025	1·074 (1·002–1·150)	0·043	1·125 (1·077–1·175)	<0·001	1·105 (1·009–1·211)	0·031	1·027 (0·965–1·093)	0·409	1·216 (1·141–1·295)	<0·001
Negative income shock less than −5%	1·051 (1·035–1·068)	<0·001	1·094 (1·064–1·125)	<0·001	1·224 (1·143–1·310)	<0·001	1·338 (1·201–1·489)	<0·001	1·067 (1·002–1·136)	0·042	1·113 (0·975–1·272)	0·114	1·043 (0·925–1·175)	0·491	1·299 (1·192–1·416)	<0·001
Negative income shock less than −10%	1·079 (1·057–1·102)	<0·001	1·130 (1·088–1·174)	<0·001	1·101 (1·000–1·213)	0·049	1·130 (0·963–1·326)	0·135	1·070 (0·983–1·166)	0·119	1·006 (0·812–1·245)	0·957	1·064 (0·906–1·249)	0·451	1·350 (1·185–1·537)	<0·001

Robust Poisson regression models were adjusted for country-fixed and year-fixed effects and the following confounding variables: rural place of residence (ref=urban), unimproved toilet facility (ref=improved), wealth index quintile (ref=q5 [richest]), maternal age at birth (years), maternal education of 5 years or less (ref=more than 5 years), maternal parity of 3 births or more (ref=less than 3 births), female child (ref=male), child age (months), child born at medical facility (ref=others). Any negative income shock (ref=no negative income shock—ie, income growth equal to or greater than zero), negative income shock less than −1% (ref=income growth −1% or more), negative income shock less than −5% (ref=income growth −5% or more), and negative income shock less than −10% (ref=income growth −10% or more). HAZ=height-for-age Z score. WHZ=weight-for-height Z score. WaSt=concurrent wasting and stunting. DBM=double burden of malnutrition. PR=prevalence ratio. Ref=reference.

* All children younger than 5 years (n=1 643 898).

† Only children aged 2–4 years (n=946 804).

The exposure to a negative income shock in the year of birth increased the prevalence of severe stunting by 6·1% (PR 1·061 [95% CI 1·045–1·076]), WaSt by 5·3% (1·053 [1·017–1·091]), and double burden of malnutrition by 4·9% (1·049 [1·006–1·093]; table 2). Income shocks in this exposure period were also associated with stunting (PR 1·027 [95% CI 1·019–1·036]), wasting (1·035 [1·014–1·056]), and overweight (1·039 [1·012–1·068]). A dose–response association according to the intensity levels of income shock in the year of birth was found for stunting (–1%: PR 1·019 [95% CI 1·011–1·027]; –5%: 1·033 [1·025–1·042]; –10%: 1·061 [1·051–1·072]) and severe stunting (–1%: 1·041 [1·026–1·055]; –5%: 1·059 [1·044–1·074]; –10%: 1·099 [1·081–1·118]).

In children aged 2–4 years, exposure to income shocks during the first 1000 days of life importantly increased the prevalence of double burden of malnutrition by 30·3% (PR 1·303 [95% CI 1·221–1·391]), obesity by 14·3% (1·143 [1·046–1·249]), and overweight by 13·8% (1·138 [1·090–1·188]; table 2). Income shocks in this period were also associated positively with stunting (PR 1·023 [95% CI 1·012–1·033]) and severe stunting (1·052 [1·033–1·072]). Dose–response associations based on the intensity of income shock in the 1000 days were observed for double burden of malnutrition (–1%: PR 1·216 [95% CI 1·141–1·295]; –5%: 1·299 [1·192–1·416]; –10%: 1·350 [1·185–1·537]) and severe stunting (–1%: 1·029 [1·010–1·049]; –5%: 1·094 [1·064–1·125]; –10%: 1·130 [1·088–1·174]).

Although no dose–response association was identified in the analyses by wealth quintile (table 3), income shocks regardless the exposure time increased significantly the prevalence of wasting (eg, q1 [poorest]: PR 1·073, [95% CI 1·025–1·122]; q5 [richest]: 0·990 [0·925–1·060] in the year of the interview), stunting (q1: 1·030 [1·014–1·046]; q5: 1·007 [0·979–1·036] in the year of birth), and WaSt (q1: 1·119 [1·049–1·195]; q5: 0·936 [0·837–1·047] in the year of birth) in the poorest quintile but not in the richest quintile. However, income shocks in the first 1000 days were associated with double burden of malnutrition in both quintiles, especially in the richest (q1: PR 1·144 [95% CI 1·016–1·289]; q5: 1·311 [1·125–1·528]). Both tests for interaction were significant for all malnutrition outcomes, reinforcing our hypothesis that income shocks operate differently by socioeconomic status (appendix p 15).

**Table 3:** Adjusted association between exposure to income shocks at different time windows and child malnutrition outcomes by wealth index quintile

	**Stunting (HAZ less than −2)**		**Severe stunting (HAZ less than −3)**		**Wasting (WHZ less than −2)**		**Severe wasting (WHZ less than −3)**		**Overweight (WHZ >2)**		**Obesity (WHZ >3)**		**WaSt (WHZ less than −2 and HAZ less than −2)**		**DBM (HAZ less than −2 and WHZ >2)**	
	PR (95% CI)	p value	PR (95% CI)	p value	PR (95% CI)	p value	PR (95% CI)	p value	PR (95% CI)	p value	PR (95% CI)	p value	PR (95% CI)	p value	PR (95% CI)	p value
Year of the interview*																
Wealth quintile 1 (poorest)	1·003 (0·986–1·020)	0·765	1·021 (0·992–1·051)	0·164	1·073 (1·025–1·122)	0·002	1·133 (1·047–1·225)	0·002	1·015 (0·949–1·086)	0·656	1·071 (0·937–1·224)	0·312	1·048 (0·971– 1·132)	0·226	1·036 (0·941–1·140)	0·471
Wealth quintile 2	1·028 (1·009–1·048)	0·004	1·077 (1·041–1·113)	<0·001	1·055 (1·004–1·108)	0·035	1·077 (0·984–1·178)	0·106	1·077 (1·006–1·154)	0·034	1·205 (1·052–1·380)	0·007	1·013 (0·930–1·103)	0·767	1·090 (0·985–1·206)	0·094
Wealth quintile 3	1·021 (0·999–1·044)	0·064	1·052 (1·011–1·095)	0·013	1·054 (0·994–1·118)	0·077	1·195 (1·069–1·337)	0·002	1·045 (0·975–1·119)	0·213	1·142 (0·995–1·312)	0·059	1·069 (0·970–1·177)	0·179	1·098 (0·984–1·224)	0·095
Wealth quintile 4	1·035 (1·010–1·062)	0·006	1·057 (1·010–1·106)	0·016	1·069 (1·007–1·133)	0·028	1·186 (1·070–1·314)	0·001	1·009 (0·945–1·077)	0·797	1·072 (0·935–1·230)	0·320	1·058 (0·945–1·183)	0·328	1·147 (1·024–1·285)	0·018
Wealth quintile 5 (richest)	0·977 (0·947–1·007)	0·136	1·010 (0·953–1·071)	0·738	0·990 (0·925–1·060)	0·780	1·051 (0·927–1·191)	0·440	1·002 (0·943–1·065)	0·943	0·992 (0·877–1·123)	0·904	0·946 (0·829–1·080)	0·410	1·003 (0·887–1·134)	0·960
Year of birth*																
Wealth quintile 1 (poorest)	1·030 (1·014–1·046)	<0·001	1·066 (1·038–1·094)	<0·001	1·078 (1·035–1·122)	<0·001	1·045 (0·972–1·124)	0·235	0·962 (0·903–1·025)	0·228	0·984 (0·867–1·118)	0·805	1·119 (1·049–1·195)	0·001	0·967 (0·887–1·053)	0·439
Wealth quintile 2	1·067 (1·049–1·085)	<0·001	1·122 (1·089–1·156)	<0·001	1·074 (1·029–1·120)	0·001	1·048 (0·970–1·132)	0·238	1·067 (1·000–1·137)	0·049	1·025 (0·904–1·162)	0·703	1·165 (1·088–1·249)	<0·001	1·144 (1·043–1·256)	0·004
Wealth quintile 3	1·048 (1·028–1·067)	<0·001	1·092 (1·056–1·128)	<0·001	1·006 (0·960–1·054)	0·799	1·058 (0·967–1·158)	0·217	0·985 (0·927–1·047)	0·638	1·038 (0·923–1·168)	0·529	0·993 (0·919–1·072)	0·852	1·017 (0·930–1·111)	0·715
Wealth quintile 4	1·052 (1·030–1·074)	<0·001	1·062 (1·023–1·103)	0·002	1·018 (0·970–1·069)	0·457	1·010 (0·927–1·101)	0·817	1·024 (0·963–1·089)	0·440	0·981 (0·874–1·102)	0·751	0·988 (0·903–1·082)	0·800	1·051 (0·953–1·159)	0·322
Wealth quintile 5 (richest)	1·007 (0·979–1·036)	0·620	1·003 (0·952–1·056)	0·910	0·991 (0·937–1·047)	0·742	0·994 (0·899–1·099)	0·903	1·078 (1·019–1·140)	0·009	1·101 (0·988–1·227)	0·080	0·936 (0·837–1·047)	0·248	1·099 (0·989–1·221)	0·080
**All years of the first 1000 days**†																
Wealth quintile 1 (poorest)	1·038 (1·018–1·057)	<0·001	1·058 (1·024–1·094)	0·001	1·055 (0·981–1·135)	0·151	1·006 (0·885–1·143)	0·931	1·093 (0·994–1·202)	0·068	0·982 (0·815–1·183)	0·845	1·048 (0·940–1·168)	0·398	1·144 (1·016–1·289)	0·026
Wealth quintile 2	1·072 (1·049–1·094)	<0·001	1·137 (1·095–1·181)	<0·001	1·054 (0·971–1·144)	0·206	0·941 (0·814–1·088)	0·410	1·241 (1·114–1·383)	<0·001	1·329 (1·071–1·649)	0·010	1·188 (1·046–1·350)	0·008	1·426 (1·238–1·644)	<0·001
Wealth quintile 3	1·054 (1·030–1·079)	<0·001	1·056 (1·012–1·101)	0·011	0·979 (0·899–1·066)	0·622	1·038 (0·893–1·207)	0·627	1·171 (1·062–1·291)	0·002	1·136 (0·940–1·373)	0·186	0·944 (0·829–1·076)	0·387	1·464 (1·278–1·678)	<0·001
Wealth quintile 4	1·062 (1·035–1·091)	<0·001	1·101 (1·049–1·156)	<0·001	1·044 (0·950–1·147)	0·371	1·076 (0·920–1·258)	0·362	1·142 (1·025–1·271)	0·016	1·029 (0·850–1·245)	0·770	1·004 (0·851–1·185)	0·963	1·405 (1·189–1·661)	<0·001
Wealth quintile 5 (richest)	1·008 (0·971–1·046)	0·665	1·025 (0·955–1·100)	0·491	1·075 (0·967–1·194)	0·181	1·110 (0·915–1·346)	0·289	1·049 (0·960–1·146)	0·290	1·179 (0·987–1·409)	0·069	0·966 (0·799–1·169)	0·723	1·311 (1·125–1·528)	0·001

Robust Poisson regression models were adjusted for country-fixed and year-fixed effects and the following confounding variables: rural place of residence (ref=urban), unimproved toilet facility (ref=improved), wealth index quintile (ref=q5 [richest]), maternal age at birth (year), maternal education of 5 years or less (ref=more than 5 years), maternal parity of 3 births or more (ref=less than 3 births), female child (ref=male), child age (month), child born at medical facility (ref=others). Any negative income shock (ref=no negative income shock—ie, income growth equal to or greater than zero). HAZ=height-for-age Z score. WHZ=weight-for-height Z score. WaSt=concurrent wasting and stunting. DBM=double burden of malnutrition. PR=prevalence ratio. Ref=reference.

* All children younger than 5 years (n=1 643 898).

† Only children aged 2–4 years (n=946 804).

In general, the main findings were supported by the sensitivity and triangulation analyses. Bivariate models, unadjusted for confounding variables (S1), the subset analyses of surveys conducted from 2000 to 2019 (S2), the analyses controlling for country-level natural disasters and armed conflicts or wars (S3), and the analyses restricting the analytical sample to only one child per household (S4) yielded similar results (table 4). Linear models using HAZ and WHZ as dependent variables produced coefficient estimates in the same direction as the main findings (appendix p 16). Income shocks at the interview year reduced WHZ (wasting), at birth reduced HAZ (stunting), and during the first 1000 days decreased HAZ and increased WHZ (overweight, obesity, and double burden of malnutrition).

**Table 4:** Sensitivity analyses of the main models assessing the association between income shocks at different exposure time windows and child malnutrition outcomes

	**Stunting (HAZ less than −2)**		**Severe stunting (HAZ less than −3)**		**Wasting (WHZ less than −2)**		**Severe wasting (WHZ less than −3)**		**Overweight (WHZ >2)**		**Obesity (WHZ >3)**		**WaSt (WHZ less than −2 and HAZ less than −2)**		**DBM (HAZ less than −2 and WHZ >2)**	
	PR (95% CI)	p value	PR (95% CI)	p value	PR (95% CI)	p value	PR (95% CI)	p value	PR (95% CI)	p value	PR (95% CI)	p value	PR (95% CI)	p value	PR (95% CI)	p value
**S1: Unadjusted for covariates**																
Year of the interview	0·977 (0·968–0·986)	<0·001	1·000 (0·984–1·017)	0·994	1·040 (1·015–1·065)	0·001	1·110 (1·063–1·159)	<0·001	1·049 (1·019–1·080)	0·001	1·102 (1·038–1·170)	0·002	1·009 (0·970–1·050)	0·653	1·040 (0·992–1·091)	0·105
Year of birth	1·016 (1·007–1·024)	<0·001	1·051 (1·035–1·067)	<0·001	1·009 (0·989–1·029)	0·383	0·998 (0·962–1·034)	0·897	1·052 (1·024–1·080)	<0·001	1·063 (1·010–1·119)	0·020	1·039 (1·004–1·076)	0·028	1·054 (1·012–1·099)	0·012
All years of the first 1000 days	1·032 (1·021–1·043)	<0·001	1·072 (1·052–1·092)	<0·001	1·033 (0·995–1·073)	0·087	1·020 (0·953–1·091)	0·569	1·145 (1·097–1·196)	<0·001	1·147 (1·048–1·256)	0·003	1·049 (0·989–1·114)	0·111	1·309 (1·227–1·397)	<0·001
**S2: Surveys from 2000 to 2019**																
Year of the interview	0·976 (0·965–0·988)	<0·001	0·995 (0·974–1·016)	0·611	1·116 (1·082–1·151)	<0·001	1·222 (1·154–1·294)	<0·001	1·044 (1·010–1·079)	0·011	1·128 (1·056–1·204)	<0·001	1·072 (1·016–1·132)	0·012	1·037 (0·981–1·095)	0·198
Year of birth	1·030 (1·020–1·040)	<0·001	1·069 (1·051–1·087)	<0·001	1·041 (1·017–1·066)	0·001	1·047 (1·004–1·093)	0·034	1·058 (1·027–1·090)	<0·001	1·063 (1·005–1·125)	0·034	1·065 (1·022–1·109)	0·003	1·055 (1·105–1·105)	0·022
All years of the first 1000 days	1·007 (0·995–1·020)	0·257	1·034 (1·010–1·058)	0·004	1·093 (1·045–1·143)	<0·001	1·063 (0·983–1·149)	0·128	1·126 (1·075–1·179)	<0·001	1·085 (0·992–1·186)	0·075	1·098 (1·017–1·186)	0·017	1·215 (1·133–1·302)	<0·001
**S3: Country-level covariates**																
Year of the interview	0·993 (0·983–1·002)	0·120	1·016 (0·999–1·033)	0·055	1·055 (1·030–1·080)	<0·001	1·134 (1·086–1·184)	<0·001	1·036 (1·007–1·067)	0·016	1·092 (1·027–1·160)	0·005	1·024 (0·984–1·066)	0·249	1·052 (1·002–1·104)	0·041
Year of birth	1·024 (1·016–1·033)	<0·001	1·058 (1·042–1·073)	<0·001	1·027 (1·006–1·048)	0·011	1·023 (0·985–1·062)	0·239	1·035 (1·007–1·064)	0·013	1·047 (0·994–1·104)	0·085	1·050 (1·014–1·087)	0·006	1·031 (0·989–1·076)	0·149
All years of the first 1000 days	1·015 (1·004–1·026)	0·005	1·045 (1·025–1·065)	<0·001	1·014 (0·976–1·053)	0·472	0·995 (0·931–1·065)	0·895	1·126 (1·076–1·177)	<0·001	1·135 (1·035–1·244)	0·007	1·013 (0·953–1·076)	0·681	1·226 (1·148–1·309)	<0·001
**S4: One child per household**																
Year of the interview	0·998 (0·987–1·009)	0·728	1·033 (1·013–1·054)	0·001	1·061 (1·023–1·093)	<0·001	1·157 (1·095–1·223)	<0·001	1·067 (1·030–1·105)	<0·001	1·179 (1·095–1·271)	<0·001	1·035 (0·984–1·088)	0·178	1·102 (1·038–1·170)	0·001
Year of birth	1·017 (1·007–1·027)	0·001	1·037 (1·019–1·055)	<0·001	1·042 (1·015–1·069)	0·002	1·021 (0·974–1·071)	0·383	1·054 (1·021–1·088)	0·001	1·091 (1·025–1·163)	0·007	1·039 (0·995–1·084)	0·086	1·064 (1·012–1·119)	0·015
All years of the first 1000 days	1·023 (1·011–1·035)	<0·001	1·056 (1·034–1·078)	<0·001	1·023 (0·982–1·067)	0·277	1·005 (0·934–1·081)	0·890	1·162 (1·109–1·216)	<0·001	1·171 (1·065–1·288)	0·001	1·024 (0·956–1·096)	0·504	1·334 (1·243–1·432)	<0·001

Robust Poisson regression models were adjusted for country-fixed and year-fixed effects. Any negative income shock (ref=no negative income shock—ie, income growth equal to or greater than zero). Analyses S2, S3, and S4 were also adjusted for the following confounding variables: rural place of residence (ref=urban), unimproved toilet facility (ref=improved), wealth index quintile (ref=q5 [richest]), maternal age at birth (year), maternal education of 5 years or less (ref=more than 5 years), maternal parity of 3 births or more (ref=less than 3 births), female child (ref=male), child age (month), child born at medical facility (ref=others). S3 was further adjusted for two country-level dummy variables indicating a national state of emergency due to a natural disaster and armed conflict or war (domestic or international). S1 and S3 analyses included the full sample: all children younger than 5 years for the year of interview and birth (n=1 643 898), and all children 2 years or older for the first 1000 days (n=946 804). S2 included only children from the surveys conducted from 2000 to 2019: children younger than 5 years for the year of interview and birth (n=1 350 016), and children 2 years or older for the first 1000 days (n=802 962). S4 included only one child (oldest) per household: children younger than 5 years for the year of interview and birth (n=1 186 948), and children 2 years or older for the first 1000 days (n=817 040). HAZ=height-for-age Z score. WHZ=weight-for-height Z score. WaSt=concurrent wasting and stunting. DBM=double burden of malnutrition. PR=prevalence ratio.

## Discussion

Our study examined the association between early life exposure to economic shocks and childhood malnutrition, using pooled data from 230 nationwide surveys across 68 LMICs, totalling more than 1·6 million children. Our findings indicate that exposure to negative income shocks can significantly increase the risk of various forms of malnutrition during childhood, including dose–response associations, and critical windows of vulnerability that vary based on the timing of economic instability and the specific type of malnutrition. Our research makes a novel contribution to the existing literature by using a disaggregated measure of subnational income shocks that accounts for the economic heterogeneity within the population of each country, and by studying malnutrition outcomes such as the double burden of malnutrition for the first time.

Income shocks in the year of the interview were mainly associated with an increased prevalence of wasting and severe wasting in children younger than 5 years. These outcomes are forms of acute undernutrition and the leading cause of early childhood mortality.^21^, ^25^ Child wasting is closely linked to short-term changes in feeding and care practices as well as recurrent infections and other illnesses.^21^ Similar studies using DHS data have also found that declines in gross national income^7^ and increases in real food price^8^ are associated with a higher risk of child wasting in LMICs. This relationship might be mediated by multiple factors, including poor dietary diversity, increased food insecurity, and a higher prevalence of infectious diseases and related symptoms, such as diarrhoea and fever,^7^ which can worsen during periods of income shocks due to limited access to food, clean water, and healthcare.^3^

Our study found dose–response associations between the intensity of income shocks at birth and child stunting outcomes. Stunting, a form of chronic undernutrition, reflects the cumulative or long-term effects of poor nutrition, including repeated episodes of wasting.^21^ We also observed that income shocks at birth were associated with an increased risk of WaSt, a life-threatening condition in which a child is both wasted and stunted.^26^ These findings suggest that economic instability during pregnancy can have profound effects on fetal and early childhood development. Potential mediators such as maternal stress, intimate partner violence, substance abuse, and undernutrition during pregnancy—factors shown to worsen in income shocks^27^, ^28^, ^29^, ^30^—can contribute to intrauterine growth restriction and low birthweight, laying the foundation for these forms of undernutrition.^14^

Exposure to negative income shocks during the first 1000 days of life (from conception to 2 years) was strongly associated with an increased prevalence of overweight, obesity, and double burden of malnutrition, in which a child is simultaneously stunted and overweight. A consistent dose–response relationship was also observed between the intensity of income shocks and double burden of malnutrition. We hypothesise that in utero and early infancy exposure to a suboptimal nutrition due to income shocks, followed by rapid postnatal catch-up growth and high-energy intake, might predispose children to overweight, obesity, or double burden of malnutrition. This complex relationship might be mediated by various biological mechanisms involved in the development of childhood obesity^31^ and double burden of malnutrition,^13^ including epigenetic alterations, dysregulation of adipose tissue development, hormonal and appetite regulation, and microbiome maturation. These effects might be further amplified by ongoing shifts in food systems across many LMICs, where ultra-processed foods and beverages are increasingly available and affordable, while physical activity levels decline.^12^

Our findings also show that income shocks generally had a greater adverse impact on undernutrition outcomes among the poorest. Wasting, stunting, and WaSt were more prevalent among the poorest households during economic shocks, highlighting the vulnerability of these populations to immediate nutritional deficits. In contrast, income shocks significantly increased the double burden of malnutrition in all wealth quintiles, especially in the richest, suggesting that wealthier households might experience different nutritional shifts in response to economic instability. These disparities underscore the complex interplay between socioeconomic status and nutritional outcomes, in which the poorest experience acute and chronic undernutrition, whereas the wealthier individuals have increased risks of overnutrition during economic crises.^32^

Our study is the first multi-country analysis to use a more granular measure of economic shock that accounts for the heterogeneity and socioeconomic inequality within each country's population. Previous studies typically relied on country–year dyad measures, such as annual growth in gross domestic product per capita, which offer limited cross-country variation when combined with individual-level data.^7^, ^9^, ^10^, ^11^ Although the WID income data used in our study can still be provisional and imperfect, they are consistent with national accounts estimates provided by the World Bank (appendix p 17). Because of difficulties in accessing data in some countries, WID income estimates can rely on imputations based on data from countries and regions with similar characteristics.^20^

Limitations associated with the wealth index used in our analysis exist. First, wealth index quintiles are constructed relative to the sample within each specific country and survey. As a result, a household in the top quintile of one country might not be as wealthy as a household in the top quintile of another country, making it difficult to directly compare wealth across countries. Additionally, we used wealth index deciles as a proxy for income deciles to approximate income distribution within a population. This approach assumes that individuals within a particular wealth decile likely correspond to the same income decile. Although this proxy might introduce some misclassification bias, it has been proposed and empirically tested as a practical method for incorporating income data when the direct income measurements are unavailable.^17^ Other studies using DHS data have already applied a similar approach.^18^, ^19^ In addition, our focus on the annual growth rate within each decile, instead of the absolute value of income, minimised potential misclassification bias.

We also acknowledge that the hierarchical structure of our data, with individuals nested in surveys and surveys nested in countries, would ideally suggest a multilevel mixed-effects approach. However, attempts to implement such models for the full dataset encountered computational challenges, including numerical overflow and model convergence failures. A pilot analysis using mixed-effects Poisson models on a smaller subset yielded estimates similar to those obtained from simpler Poisson regression models, supporting the validity of our current approach (appendix p 20).

In conclusion, our study highlights that income shocks can significantly increase the risk of various forms of childhood malnutrition, with critical periods of vulnerability that vary by the timing of economic instability and the type of malnutrition. Policymakers and public health practitioners must recognise these critical periods and develop targeted interventions to safeguard maternal and child nutrition, particularly during times of economic hardship. Addressing the root causes of poverty and ensuring economic stability for the most vulnerable are essential steps toward mitigating the long-term impacts of the polycrisis and income shocks on child health and nutrition. Additionally, tailored strategies are needed to address all forms of malnutrition across different socioeconomic groups, ensuring that all children have the opportunity to achieve optimal growth and development.

### Contributors

### Data sharing

All data used in this study are from publicly available sources: Demographic and Health Survey (DHS) program, World Inequality Database, World Bank, and Varieties of Democracy. However, DHS datasets can only be shared with other researchers with a written consent of the DHS Program (https://dhsprogram.com/data/terms-of-use.cfm). Analysis files and other datasets can be shared upon a reasonable request to the corresponding author.

## Declaration of interests

We declare no competing interests.
