# Supplementary material for: Early life exposure to economic shocks and association with childhood malnutrition: a pooled analysis of 230 nationwide surveys from 68 low-income and middle-income countries
Source: Lancet Glob Health. 2025 Jun 27;13(8):e1367–77. doi: 10.1016/S2214-109X(25)00153-6 (PMC12284386; doi:10.1016/S2214-109X(25)00153-6)
Supplement: Supplementary appendix [file mmc1.pdf]

THE LANCET
Global Health

## Supplementary appendix

This appendix formed part of the original submission and has been peer reviewed. We post it as supplied by the authors.

Supplement to: Silva NJ, Paixão ES, Brachowicz N, et al. Early life exposure to economic shocks and association with childhood malnutrition: a pooled analysis of 230 nationwide surveys from 68 low-income and middle-income countries. *Lancet Glob Health* 2025; published online June 27. https://doi.org/10.1016/S2214-109X(25)00153-6.

# Supplementary Material

**Early-life exposure to economic shocks and multiple forms of childhood malnutrition: a pooled analysis of 230 nationwide surveys from 68 low- and middle-income countries**

Natanael J Silva, MSc · Enny S Paixão, PhD · Nicolai Brachowicz, MSc · Gonzalo Barreix, MSc · Elisa L Basterra, MSc· Felipe A Rubio, PhD · Delia Boccia, PhD · Rita C Ribeiro-Silva, PhD · Mauricio L Barreto, MD · Ivalda Macicame, MD· Aliya Naheed, MD · Denise Naniche, PhD · Davide Rasella, PhD

Appendix 1: Details on the calculation of anthropometric z-scores
Appendix 2: Methodological aspects of the DHS-WID data linkage
Appendix 3: Flowchart of the final study sample selection. DHS, 1990-2022
Appendix 4: Summary descriptive by region and survey included in the study. DHS, 1990-2022
Appendix 5: Prevalence of childhood malnutrition outcomes by region and country based on the most recent survey
Appendix 6: Frequency of children exposed to any income shocks at different exposure windows by region and country
Appendix 7: Full models of the association between exposure to income shocks and child malnutrition
Appendix 8: Models with interaction term between income shock and wealth quintiles
Appendix 9: Adjusted models with HAZ and WHZ as dependent continuous variables
Appendix 10: Consistency check between pretax income from the WID and national accounts from the World Bank
Appendix 11: Pilot analyses with multilevel mixed-effects Poisson regression and simple Poisson regression models

**Appendix 1: Details on the calculation of anthropometric z-scores**

Height-for-age z-scores (HAZ) and weight-for-height z-scores (WHZ) were recalculated for all surveys in accordance with the Guide to DHS Statistics.[1] For this calculation, we used height (cm), weight (kg), age (in days), and sex information for all children aged 0-59 months from the DHS children's recode files, which include a record for every child born to interviewed women in the five years preceding the survey. The z-scores were derived using the WHO Child Growth Standards curves and the WHO macro package 'igrowup' for Stata.[2,3] To address missing or unknown birth dates, the DHS Program employs the following imputation methods: in DHS-VI and earlier rounds, the 15th day of the month is assigned as the date of birth; in DHS-VII and later rounds, the day of birth is randomly imputed based on the construction of logical ranges, which are refined in three steps resulting in successively narrower or constrained ranges.[4] For our study, observations with missing data or biologically implausible values for height and weight were excluded. Biologically implausible values were defined as HAZ < -6 or > 6 and WHZ < -6 or > 5.[5]

References:

1. https://dhsprogram.com/data/Guide-to-DHS-Statistics/index.htm#t=Nutritional_Status.htm
2. De Onis M, Onyango AW, Borghi E, Siyam A, Nishida C, Siekmann J. Development of a WHO growth reference for school-aged children and adolescents. Bull World Health Organ. 2007 Sep;85(9):660-7.
3. https://www.who.int/tools/child-growth-standards/software
4. https://dhsprogram.com/pubs/pdf/DHSG3/DHS_Data_Editing.pdf
5. World Health Organization, United Nations Children's Fund. Recommendations for data collection, analysis and reporting on anthropometric indicators in children under 5 years old. Geneva: WHO, UNICEF; 2019.

**Appendix 2: Methodological aspects of the DHS-WID data linkage**

This study integrates cross-sectional individual data from the Demographic and Health Surveys (DHS, 1990-2022) with national-level longitudinal economic data from the World Inequality Database (WID, 1984-2022). From the DHS, we obtained data on child nutrition, pregnancy, maternal characteristics, and household conditions. From the WID, we extracted annual estimates of pretax national income by income decile to measure economic shocks. The integration of these datasets was conducted in three main steps.

Step 1: The DHS wealth index

DHS are nationally representative household surveys that provide accurate data on demographics and health. DHS surveys do not collect direct income or expenditure data but provide a wealth index, which is widely used to assess economic inequalities across household.[1]

The wealth index is constructed using household asset data through principal components analysis (PCA), accounting for differences in the relative importance of assets between urban and rural households. The variables included in the calculation of the score were household assets (e.g., cookstove, bicycle, car), building materials of the house (e.g., wood flooring, brick walls, corrugated roofing), and access to utilities (e.g., sanitation, electricity). This wealth score index is provided with the original survey datasets and is calculated following a standardized methodology.[1]

For our study, we ranked households in each survey based on the resulting wealth score index and divided into ten equally sized groups (deciles) and five equally sized groups (quintiles). Wealth deciles were used to match DHS data with income data from the WID, as further detailed, while wealth quintiles were used as covariate to control for socioeconomic confounders in the models.

Step 2: The WID income data

The WID is an extensive and open database on the world distribution of income and wealth, both between and within countries. It provides annual estimates of income and wealth distribution by collecting and harmonizing data from various sources, including national household surveys, national accounts, tax records, and administrative data. For countries with missing or incomplete data, the WID uses statistical models to estimate distributions based on existing trends and data from similar nations. The data is carefully validated, cross-checked with alternative sources, and adjusted for biases. The methods and concepts used in the WID are extensively documented elsewhere.[2]

For this study, we obtained annual estimates of pretax national income for each decile of the country's income distribution. This income measure is the WID benchmark distributional income concept. It includes social insurance benefits (and remove corresponding contributions), but exclude other forms of redistribution (income tax, social assistance benefits, etc.).[2] To access and download country-year income data and US$ PPP conversion factors, we used the WID macro package 'wid' available in Stata.

Step 3: DHS and WID linkage approach

To incorporate income data from WID into DHS, we employed a proxy ranking approach based on the assumption that households in the same wealth decile are similarly positioned in the income decile. This approach has been validated in previous studies where direct income measurements were unavailable.[3,4]

To operationally link the two datasets, we used three key variables: country, year, and wealth/income deciles. Since our study includes three definitions of temporal exposure, we performed a total of five merges to match each of the years of interest: year of the interview, year of birth, and all years within the first 1,000 days (i.e. year of conception, year of birth, year of 1st birthday, and year of 2nd birthday). A pregnancy period of nine months was considered to compute the year of conception based on the child's date of birth.

It is worth noting that this approach may have limitations. Since the assets used in wealth index were measured at the time of the interview, households may not have been in the same wealth decile in the years to the interview, which could bias the determination of income during the preceding time windows of birth and the first 1,000 days. However, while minor asset shifts may occur, the wealth index represents a more structural socio-economic status and unlikely to significantly alter households' decile positioning.[5]

References:

1. Croft TN, Allen CK, Zachary BW, et al. Guide to DHS Statistics. Rockville, MD: ICF; 2023.

2. Blanchet T, Chancel L, Flores I, Morgan M, Alvaredo F, Atkinson AB, Bauluz L, Fisher-Post M, Garbinti B, Goupille-Lebret J, Martínez-Toledano C. Distributional National Accounts Guidelines. Methods and Concepts used in the World Inequality Database. Paris: World Inequality Lab. 2024.
3. Harttgen K, Vollmer S. Using an asset index to simulate household income. Econ Lett. 2013;121(2):257–262.
4. Fink G, Victora CG, Harttgen K, Vollmer S, Vidaletti LP, Barros AJ. Measuring socioeconomic inequalities with predicted absolute incomes rather than wealth quintiles: a comparative assessment using child stunting data from national surveys. Am J Public Health. 2017 Apr;107(4):550-555.
5. Rutstein SO, Johnson K. The DHS Wealth Index. DHS Comparative Reports No. 6. Calverton, MD: ORC Macro; 2004.

**Appendix 3: Flowchart of the final study sample selection. DHS, 1990-2022**

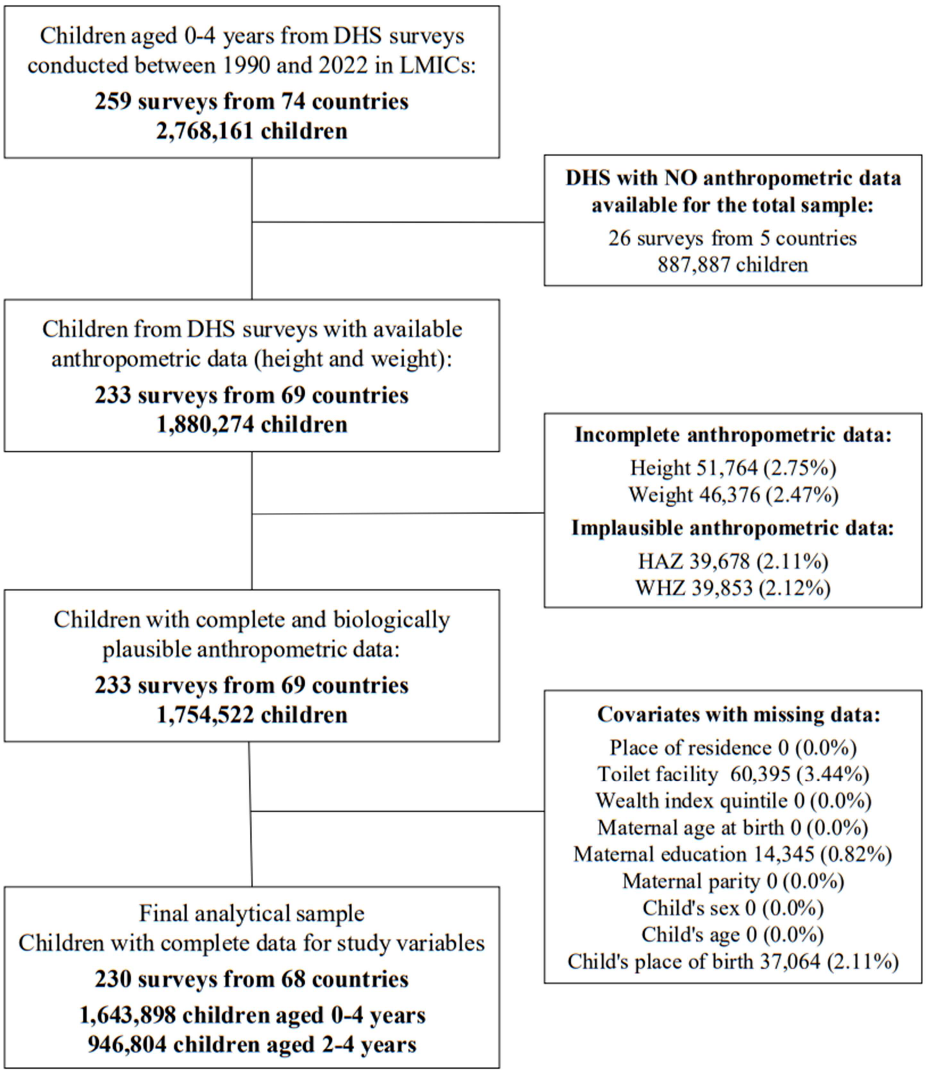

**Appendix 4: Summary descriptive by region and survey included in the study. DHS, 1990-2022**

| Region and country names | Country code | Start year of the survey | Number of households | Number of mothers | Number of children |
|---|---|---|---|---|---|
| **South Asia** | | | **428,454** | **448,895** | **566,341** |
| Bangladesh | BGD | 1996 | 3,446 | 3,615 | 4,425 |
| Bangladesh | BGD | 2014 | 3,572 | 3,664 | 3,793 |
| Bangladesh | BGD | 2007 | 3,932 | 4,053 | 4,787 |
| Bangladesh | BGD | 2011 | 5,824 | 6,038 | 6,945 |
| Bangladesh | BGD | 2017 | 3,922 | 4,013 | 4,176 |
| Bangladesh | BGD | 2004 | 4,516 | 4,812 | 5,824 |
| Bangladesh | BGD | 1999 | 3,982 | 4,195 | 5,113 |
| India | IND | 2019 | 144,271 | 150,013 | 188,894 |
| India | IND | 2015 | 158,720 | 165,830 | 215,473 |
| India | IND | 2005 | 28,382 | 29,569 | 38,844 |
| India | IND | 1992 | 20,394 | 22,138 | 26,928 |
| India | IND | 1998 | 21,557 | 22,784 | 25,079 |
| Maldives | MDV | 2016 | 1,773 | 2,053 | 2,320 |
| Maldives | MDV | 2009 | 1,810 | 2,058 | 2,300 |
| Nepal | NPL | 2011 | 1,694 | 1,764 | 2,214 |
| Nepal | NPL | 2021 | 1,247 | 1,287 | 1,378 |
| Nepal | NPL | 1996 | 3,162 | 3,386 | 3,734 |
| Nepal | NPL | 2001 | 4,000 | 4,260 | 5,884 |
| Nepal | NPL | 2016 | 1,715 | 1,804 | 2,186 |
| Nepal | NPL | 2006 | 3,522 | 3,745 | 4,963 |
| Pakistan | PAK | 2017 | 2,400 | 2,761 | 3,935 |
| Pakistan | PAK | 2012 | 1,867 | 2,064 | 2,953 |
| Pakistan | PAK | 1990 | 2,746 | 2,989 | 4,193 |
| **West and Central Africa** | | | **233,859** | **272,412** | **368,098** |
| Benin | BEN | 2001 | 2,717 | 3,141 | 4,288 |
| Benin | BEN | 2006 | 8,033 | 8,917 | 12,258 |
| Benin | BEN | 1996 | 1,970 | 2,348 | 2,539 |
| Benin | BEN | 2011 | 6,335 | 6,848 | 9,087 |
| Benin | BEN | 2017 | 7,159 | 8,272 | 11,738 |
| Burkina Faso | BFA | 2010 | 4,063 | 4,815 | 6,484 |
| Burkina Faso | BFA | 2003 | 4,908 | 6,254 | 8,012 |
| Burkina Faso | BFA | 1998 | 2,658 | 3,361 | 4,440 |
| Burkina Faso | BFA | 2021 | 2,764 | 3,157 | 3,351 |
| Burkina Faso | BFA | 1992 | 2,659 | 3,316 | 4,390 |
| Cameroon | CMR | 2011 | 3,036 | 3,413 | 4,859 |
| Cameroon | CMR | 1998 | 1,372 | 1,544 | 1,706 |
| Cameroon | CMR | 1991 | 1,454 | 1,739 | 2,562 |
| Cameroon | CMR | 2004 | 1,945 | 2,184 | 3,050 |
| Cameroon | CMR | 2018 | 2,534 | 2,925 | 4,202 |
| Central African Republic | CAF | 1994 | 1,855 | 2,099 | 2,351 |
| Chad | TCD | 2004 | 2,717 | 3,002 | 4,326 |
| Chad | TCD | 2014 | 6,088 | 6,561 | 9,885 |
| Chad | TCD | 1996 | 3,491 | 3,993 | 5,674 |
| Congo | COG | 2005 | 2,671 | 2,938 | 3,730 |
| Congo | COG | 2011 | 2,850 | 3,089 | 4,261 |
| Côte d'Ivoire | CIV | 1998 | 906 | 1,135 | 1,435 |
| Côte d'Ivoire | CIV | 2021 | 2,439 | 2,679 | 2,924 |
| Côte d'Ivoire | CIV | 1994 | 756 | 950 | 1,031 |
| Côte d'Ivoire | CIV | 2011 | 2,033 | 2,372 | 3,132 |
| Democratic Republic of the Congo | COD | 2007 | 2,080 | 2,222 | 3,202 |
| Democratic Republic of the Congo | COD | 2013 | 4,893 | 5,211 | 7,967 |
| Gabon | GAB | 2000 | 1,936 | 2,310 | 3,206 |
| Gabon | GAB | 2019 | 3,418 | 3,792 | 5,148 |
| Gabon | GAB | 2012 | 2,109 | 2,415 | 3,268 |
| Gambia | GMB | 2019 | 1,812 | 2,677 | 3,648 |
| Gambia | GMB | 2013 | 1,541 | 2,190 | 3,062 |
| Ghana | GHA | 1998 | 2,022 | 2,135 | 2,769 |
| Ghana | GHA | 2008 | 1,729 | 1,816 | 2,355 |
| Ghana | GHA | 2014 | 1,940 | 2,029 | 2,659 |
| Ghana | GHA | 2003 | 2,199 | 2,330 | 3,030 |
| Ghana | GHA | 1993 | 1,688 | 1,769 | 1,897 |
| Guinea | GIN | 2018 | 2,147 | 2,498 | 3,321 |
| Guinea | GIN | 2005 | 1,618 | 1,963 | 2,566 |

| | | | | | |
|---|---|---|---|---|---|
| Guinea | GIN | 1999 | 2,698 | 3,356 | 4,269 |
| Guinea | GIN | 2012 | 1,948 | 2,337 | 3,044 |
| Liberia | LBR | 2013 | 2,116 | 2,366 | 3,132 |
| Liberia | LBR | 2019 | 1,798 | 1,927 | 2,391 |
| Liberia | LBR | 2006 | 2,946 | 3,254 | 4,260 |
| Mali | MLI | 2018 | 5,198 | 5,806 | 8,439 |
| Mali | MLI | 1995 | 3,827 | 4,295 | 4,691 |
| Mali | MLI | 2012 | 2,771 | 3,087 | 4,332 |
| Mali | MLI | 2006 | 6,874 | 7,779 | 10,809 |
| Mali | MLI | 2001 | 5,943 | 6,738 | 9,200 |
| Mauritania | MRT | 2019 | 5,697 | 6,608 | 9,475 |
| Niger | NER | 2012 | 2,790 | 3,204 | 4,788 |
| Niger | NER | 1998 | 3,019 | 3,556 | 3,907 |
| Niger | NER | 2006 | 2,182 | 2,619 | 3,652 |
| Nigeria | NGA | 1990 | 3,624 | 4,129 | 5,859 |
| Nigeria | NGA | 2013 | 15,512 | 17,246 | 24,316 |
| Nigeria | NGA | 2018 | 7,069 | 7,769 | 11,084 |
| Nigeria | NGA | 2008 | 12,407 | 13,773 | 18,964 |
| Nigeria | NGA | 2003 | 2,778 | 3,092 | 4,292 |
| Sao Tome and Principe | STP | 2008 | 1,137 | 1,167 | 1,464 |
| Senegal | SEN | 2019 | 2,695 | 3,959 | 5,335 |
| Senegal | SEN | 2015 | 2,814 | 4,256 | 5,931 |
| Senegal | SEN | 2012 | 2,640 | 3,964 | 5,712 |
| Senegal | SEN | 2018 | 2,822 | 4,276 | 5,828 |
| Senegal | SEN | 2010 | 1,804 | 2,615 | 3,630 |
| Senegal | SEN | 2017 | 5,010 | 7,516 | 10,294 |
| Senegal | SEN | 2005 | 1,391 | 1,959 | 2,751 |
| Sierra Leone | SLE | 2008 | 1,440 | 1,585 | 1,993 |
| Sierra Leone | SLE | 2019 | 2,897 | 3,197 | 4,051 |
| Sierra Leone | SLE | 2013 | 2,872 | 3,250 | 4,070 |
| Togo | TGO | 1998 | 2,491 | 2,956 | 3,188 |
| Togo | TGO | 2013 | 2,104 | 2,362 | 3,134 |
| **Eastern and Southern Africa** | | | **218,239** | **227,977** | **302,559** |
| Angola | AGO | 2015 | 3,963 | 4,169 | 6,251 |
| Burundi | BDI | 2010 | 2,269 | 2,313 | 3,432 |
| Burundi | BDI | 2016 | 4,095 | 4,145 | 5,973 |
| Comoros | COM | 2012 | 1,618 | 1,659 | 2,360 |
| Comoros | COM | 1996 | 805 | 824 | 961 |
| Eswatini | SWZ | 2006 | 1,418 | 1,604 | 1,985 |
| Ethiopia | ETH | 2000 | 6,129 | 6,233 | 8,553 |
| Ethiopia | ETH | 2019 | 3,623 | 3,676 | 5,057 |
| Ethiopia | ETH | 2010 | 6,651 | 6,769 | 9,457 |
| Ethiopia | ETH | 2016 | 6,247 | 6,328 | 8,724 |
| Ethiopia | ETH | 2005 | 2,716 | 2,741 | 3,756 |
| Kenya | KEN | 2022 | 9,197 | 9,360 | 10,406 |
| Kenya | KEN | 2008 | 3,478 | 3,607 | 5,049 |
| Kenya | KEN | 1998 | 2,510 | 2,616 | 2,925 |
| Kenya | KEN | 2014 | 13,235 | 13,549 | 18,283 |
| Kenya | KEN | 2003 | 3,214 | 3,322 | 4,628 |
| Lesotho | LSO | 2009 | 1,218 | 1,280 | 1,561 |
| Lesotho | LSO | 2014 | 1,017 | 1,055 | 1,237 |
| Lesotho | LSO | 2004 | 1,070 | 1,124 | 1,338 |
| Madagascar | MDG | 2021 | 4,316 | 4,454 | 5,699 |
| Madagascar | MDG | 2003 | 3,113 | 3,214 | 4,395 |
| Madagascar | MDG | 1997 | 2,616 | 2,713 | 3,022 |
| Malawi | MWI | 2010 | 3,272 | 3,368 | 4,533 |
| Malawi | MWI | 2004 | 5,860 | 5,998 | 8,014 |
| Malawi | MWI | 1992 | 2,216 | 2,324 | 3,203 |
| Malawi | MWI | 2000 | 6,495 | 6,708 | 8,970 |
| Malawi | MWI | 2015 | 4,035 | 4,141 | 5,074 |
| Mozambique | MOZ | 2011 | 6,343 | 6,725 | 9,183 |
| Mozambique | MOZ | 2003 | 5,329 | 5,880 | 7,923 |
| Mozambique | MOZ | 1997 | 2,938 | 3,166 | 3,368 |
| Namibia | NAM | 1992 | 1,594 | 1,996 | 2,560 |
| Namibia | NAM | 2006 | 2,674 | 2,950 | 3,567 |
| Namibia | NAM | 2013 | 1,321 | 1,471 | 1,751 |
| Namibia | NAM | 2000 | 2,012 | 2,266 | 2,807 |
| Rwanda | RWA | 2014 | 2,692 | 2,732 | 3,507 |
| Rwanda | RWA | 1992 | 2,992 | 3,039 | 4,210 |

| | | | | | |
|---|---|---|---|---|---|
| Rwanda | RWA | 2005 | 2,452 | 2,493 | 3,632 |
| Rwanda | RWA | 2010 | 2,966 | 3,014 | 4,047 |
| Rwanda | RWA | 2019 | 2,872 | 2,926 | 3,725 |
| Rwanda | RWA | 2000 | 4,210 | 4,260 | 6,024 |
| South Africa | ZAF | 2016 | 847 | 923 | 1,032 |
| Tanzania | TZA | 2004 | 4,613 | 4,911 | 6,892 |
| Tanzania | TZA | 1996 | 3,504 | 3,829 | 5,140 |
| Tanzania | TZA | 2015 | 5,754 | 6,187 | 8,503 |
| Tanzania | TZA | 1999 | 1,575 | 1,710 | 2,369 |
| Tanzania | TZA | 2009 | 4,407 | 4,684 | 6,548 |
| Uganda | UGA | 2000 | 3,284 | 3,379 | 4,963 |
| Uganda | UGA | 2006 | 1,452 | 1,513 | 2,295 |
| Uganda | UGA | 1995 | 3,265 | 3,472 | 4,572 |
| Uganda | UGA | 2016 | 2,915 | 3,030 | 4,274 |
| Uganda | UGA | 2011 | 1,319 | 1,374 | 2,039 |
| Zambia | ZMB | 1996 | 3,594 | 3,949 | 5,502 |
| Zambia | ZMB | 2001 | 3,612 | 3,824 | 5,287 |
| Zambia | ZMB | 2007 | 3,385 | 3,546 | 5,017 |
| Zambia | ZMB | 2018 | 6,151 | 6,552 | 8,443 |
| Zambia | ZMB | 2013 | 7,856 | 8,264 | 11,182 |
| Zimbabwe | ZWE | 1999 | 1,988 | 2,091 | 2,547 |
| Zimbabwe | ZWE | 2005 | 2,985 | 3,154 | 3,825 |
| Zimbabwe | ZWE | 2015 | 3,761 | 3,910 | 4,688 |
| Zimbabwe | ZWE | 1994 | 1,816 | 1,944 | 2,093 |
| Zimbabwe | ZWE | 2010 | 3,365 | 3,519 | 4,198 |
| **Latin America and Caribbean** | | | **178,890** | **186,101** | **228,793** |
| Bolivia | BOL | 2003 | 6,465 | 6,665 | 9,023 |
| Bolivia | BOL | 1993 | 2,497 | 2,551 | 2,904 |
| Bolivia | BOL | 1998 | 4,246 | 4,356 | 6,189 |
| Brazil | BRA | 1996 | 3,138 | 3,216 | 4,064 |
| Colombia | COL | 1995 | 3,328 | 3,479 | 4,520 |
| Colombia | COL | 2000 | 3,150 | 3,284 | 3,284 |
| Colombia | COL | 2004 | 9,635 | 10,032 | 10,032 |
| Colombia | COL | 2009 | 12,703 | 13,209 | 13,209 |
| Dominican Republic | DOM | 1996 | 2,549 | 2,620 | 3,641 |
| Dominican Republic | DOM | 2007 | 6,944 | 7,132 | 8,938 |
| Dominican Republic | DOM | 2002 | 6,507 | 6,637 | 8,910 |
| Dominican Republic | DOM | 2013 | 2,471 | 2,543 | 3,078 |
| Guatemala | GTM | 2014 | 8,550 | 9,064 | 11,576 |
| Guatemala | GTM | 1998 | 2,456 | 2,581 | 3,883 |
| Guatemala | GTM | 1995 | 5,282 | 5,555 | 8,527 |
| Haiti | HTI | 2016 | 4,137 | 4,392 | 5,449 |
| Haiti | HTI | 2000 | 3,628 | 3,846 | 5,480 |
| Haiti | HTI | 1994 | 1,421 | 1,533 | 2,227 |
| Haiti | HTI | 2005 | 1,604 | 1,701 | 2,287 |
| Haiti | HTI | 2012 | 2,883 | 3,069 | 3,888 |
| Honduras | HND | 2005 | 6,760 | 7,041 | 9,040 |
| Honduras | HND | 2011 | 7,506 | 7,855 | 9,562 |
| Nicaragua | NIC | 2001 | 4,161 | 4,435 | 5,809 |
| Nicaragua | NIC | 1997 | 4,615 | 4,974 | 6,789 |
| Paraguay | PRY | 1990 | 2,277 | 2,396 | 3,622 |
| Peru | PER | 2011 | 6,920 | 7,118 | 8,492 |
| Peru | PER | 2005 | 8,086 | 8,329 | 8,329 |
| Peru | PER | 2009 | 7,426 | 7,658 | 9,170 |
| Peru | PER | 2000 | 8,537 | 8,797 | 8,797 |
| Peru | PER | 2010 | 6,934 | 7,113 | 8,488 |
| Peru | PER | 1996 | 10,653 | 11,148 | 14,879 |
| Peru | PER | 1991 | 4,080 | 4,241 | 5,765 |
| Peru | PER | 2012 | 7,341 | 7,531 | 8,942 |
| **Middle East and North Africa** | | | **66,491** | **69,590** | **97,254** |
| Egypt | EGY | 1995 | 6,713 | 7,349 | 10,382 |
| Egypt | EGY | 2005 | 8,270 | 8,775 | 11,741 |
| Egypt | EGY | 2000 | 6,822 | 7,277 | 9,983 |
| Egypt | EGY | 2003 | 4,014 | 4,265 | 5,781 |
| Egypt | EGY | 2008 | 6,669 | 6,985 | 9,141 |
| Egypt | EGY | 2014 | 9,820 | 9,963 | 13,144 |
| Jordan | JOR | 1990 | 3,832 | 3,971 | 6,650 |
| Jordan | JOR | 2012 | 4,046 | 4,086 | 6,048 |
| Jordan | JOR | 2002 | 3,141 | 3,166 | 4,670 |

| | | | | | |
|---|---|---|---|---|---|
| Jordan | JOR | 1997 | 3,364 | 3,473 | 5,613 |
| Jordan | JOR | 2007 | 2,812 | 2,833 | 4,268 |
| Morocco | MAR | 1992 | 2,963 | 3,191 | 4,542 |
| Morocco | MAR | 2003 | 4,025 | 4,256 | 5,291 |
| **Eastern Europe and Central Asia** | | | **34,995** | **36,750** | **46,553** |
| Albania | ALB | 2008 | 1,109 | 1,123 | 1,305 |
| Albania | ALB | 2017 | 2,045 | 2,071 | 2,406 |
| Armenia | ARM | 2000 | 1,127 | 1,162 | 1,493 |
| Armenia | ARM | 2005 | 957 | 982 | 1,216 |
| Armenia | ARM | 2015 | 1,211 | 1,246 | 1,519 |
| Armenia | ARM | 2010 | 1,029 | 1,067 | 1,329 |
| Azerbaijan | AZE | 2006 | 1,419 | 1,464 | 1,901 |
| Kazakhstan | KAZ | 1999 | 443 | 453 | 548 |
| Kazakhstan | KAZ | 1995 | 641 | 656 | 737 |
| Kyrgyzstan | KGZ | 1997 | 832 | 880 | 973 |
| Kyrgyzstan | KGZ | 2012 | 2,739 | 2,851 | 3,836 |
| Moldova | MDA | 2005 | 1,125 | 1,129 | 1,259 |
| Tajikistan | TJK | 2017 | 3,350 | 3,869 | 5,488 |
| Tajikistan | TJK | 2012 | 2,791 | 3,201 | 4,439 |
| Turkey | TUR | 2003 | 2,896 | 2,985 | 3,905 |
| Turkey | TUR | 2013 | 2,166 | 2,196 | 2,671 |
| Turkey | TUR | 1993 | 2,383 | 2,474 | 3,132 |
| Turkey | TUR | 1998 | 2,123 | 2,226 | 2,772 |
| Turkey | TUR | 2008 | 2,150 | 2,193 | 2,656 |
| Turkey | TUR | 2018 | 1,632 | 1,650 | 1,992 |
| Uzbekistan | UZB | 1996 | 827 | 872 | 976 |
| **East Asia and Pacific** | | | **26,020** | **26,707** | **34,300** |
| Cambodia | KHM | 2005 | 2,714 | 2,769 | 3,557 |
| Cambodia | KHM | 2014 | 3,530 | 3,645 | 4,297 |
| Cambodia | KHM | 2021 | 2,017 | 2,040 | 2,137 |
| Cambodia | KHM | 2000 | 2,625 | 2,658 | 3,501 |
| Cambodia | KHM | 2010 | 2,900 | 2,994 | 3,663 |
| Myanmar | MMR | 2015 | 3,308 | 3,397 | 4,088 |
| Timor-Leste | TLS | 2016 | 3,918 | 4,054 | 5,490 |
| Timor-Leste | TLS | 2009 | 5,008 | 5,150 | 7,567 |
| **Global** | | | **1,186,948** | **1,268,432** | **1,643,898** |

**Appendix 5: Prevalence of childhood malnutrition outcomes by region and country based on the most recent survey. DHS, 1990-2022**

| Region and country names | Country code | Year | Stunting (HAZ<-2) | Severe stunting (HAZ<-3) | Wasting (WHZ<-2) | Severe wasting (WHZ<-3) | Overweight (WHZ>2) | Obesity (WHZ>3) | WaSt (WHZ<-2 & HAZ<-2) | DBM (HAZ<-2 & WHZ>2) |
|---|---|---|---|---|---|---|---|---|---|---|
| **South Asia** | | | **35.4** | **15.0** | **17.4** | **7.0** | **3.7** | **1.4** | **4.5** | **2.2** |
| Bangladesh | BGD | 2017 | 31.2 | 9.0 | 8.4 | 1.5 | 1.9 | 0.6 | 2.9 | 0.6 |
| India | IND | 2019 | 35.6 | 15.2 | 18.1 | 7.3 | 3.7 | 1.4 | 4.6 | 2.3 |
| Maldives | MDV | 2016 | 15.3 | 4.2 | 9.1 | 2.0 | 4.8 | 1.7 | 1.5 | 1.0 |
| Nepal | NPL | 2021 | 27.9 | 6.5 | 6.6 | 0.7 | 1.2 | 0.2 | 2.7 | 0.2 |
| Pakistan | PAK | 2017 | 38.3 | 18.8 | 7.9 | 3.1 | 3.0 | 0.9 | 2.9 | 1.7 |
| **West and Central Africa** | | | **30.9** | **12.9** | **8.1** | **2.3** | **3.1** | **0.8** | **2.8** | **1.4** |
| Benin | BEN | 2017 | 32.7 | 11.8 | 5.2 | 1.1 | 1.8 | 0.3 | 2.0 | 0.7 |
| Burkina Faso | BFA | 2021 | 21.5 | 6.6 | 9.6 | 2.0 | 1.6 | 0.4 | 2.5 | 0.9 |
| Cameroon | CMR | 2018 | 27.8 | 12.6 | 4.0 | 1.4 | 10.7 | 2.8 | 1.1 | 3.9 |
| Central African Republic | CAF | 1994 | 39.7 | 18.5 | 9.2 | 2.8 | 3.8 | 1.0 | 3.5 | 2.0 |
| Chad | TCD | 2014 | 43.0 | 23.9 | 14.4 | 4.6 | 2.5 | 0.8 | 5.9 | 1.4 |
| Congo | COG | 2011 | 27.3 | 9.9 | 5.4 | 1.6 | 3.5 | 0.9 | 1.3 | 1.5 |
| Côte d'Ivoire | CIV | 2021 | 23.3 | 7.5 | 8.2 | 2.4 | 2.7 | 0.5 | 2.2 | 0.9 |
| Democratic Republic of the Congo | COD | 2013 | 43.6 | 22.8 | 8.0 | 2.9 | 4.1 | 1.3 | 2.5 | 2.5 |
| Gabon | GAB | 2019 | 20.6 | 7.5 | 3.4 | 0.8 | 5.1 | 1.2 | 0.8 | 1.6 |
| Gambia | GMB | 2019 | 18.1 | 3.4 | 5.1 | 0.5 | 1.8 | 0.1 | 1.2 | 0.4 |
| Ghana | GHA | 2014 | 19.4 | 5.5 | 4.9 | 0.9 | 2.4 | 0.5 | 1.5 | 0.8 |
| Guinea | GIN | 2018 | 30.4 | 12.6 | 8.7 | 3.3 | 5.5 | 1.7 | 1.8 | 3.1 |
| Liberia | LBR | 2019 | 31.6 | 10.3 | 4.2 | 0.8 | 4.2 | 0.6 | 1.6 | 1.5 |
| Mali | MLI | 2018 | 27.3 | 11.0 | 9.5 | 2.8 | 2.3 | 0.8 | 3.0 | 1.3 |
| Mauritania | MRT | 2019 | 25.9 | 8.6 | 6.6 | 1.0 | 1.5 | 0.2 | 2.0 | 0.4 |
| Niger | NER | 2012 | 40.8 | 19.3 | 18.9 | 7.2 | 2.7 | 1.2 | 7.6 | 1.7 |
| Nigeria | NGA | 2018 | 36.0 | 16.5 | 6.7 | 1.7 | 1.9 | 0.3 | 3.1 | 0.8 |
| São Tomé and Príncipe | STP | 2008 | 29.2 | 12.7 | 12.3 | 4.6 | 11.6 | 4.6 | 2.1 | 6.2 |
| Senegal | SEN | 2019 | 19.3 | 5.4 | 8.9 | 1.5 | 1.5 | 0.4 | 2.6 | 0.4 |
| Sierra Leone | SLE | 2019 | 30.3 | 11.4 | 5.6 | 1.2 | 4.7 | 1.0 | 1.8 | 2.3 |
| Togo | TGO | 2013 | 28.2 | 9.4 | 7.4 | 1.8 | 2.0 | 0.5 | 2.4 | 0.9 |
| **Eastern and Southern Africa** | | | **33.6** | **12.0** | **5.1** | **1.3** | **4.7** | **1.2** | **1.7** | **2.0** |
| Angola | AGO | 2015 | 38.2 | 15.4 | 5.1 | 1.3 | 3.5 | 0.9 | 2.0 | 1.7 |
| Burundi | BDI | 2016 | 54.2 | 23.5 | 5.0 | 0.8 | 1.5 | 0.4 | 3.3 | 0.8 |
| Comoros | COM | 2012 | 28.6 | 13.8 | 11.5 | 4.4 | 9.8 | 4.5 | 2.0 | 5.4 |
| Eswatini | SWZ | 2006 | 27.4 | 9.4 | 2.5 | 0.8 | 11.2 | 3.3 | 0.5 | 3.6 |
| Ethiopia | ETH | 2019 | 35.7 | 13.4 | 9.2 | 1.8 | 1.8 | 0.2 | 3.8 | 0.6 |
| Kenya | KEN | 2022 | 17.8 | 4.3 | 6.8 | 0.9 | 2.8 | 0.4 | 1.8 | 0.5 |
| Lesotho | LSO | 2014 | 34.1 | 11.3 | 3.6 | 1.1 | 8.1 | 2.4 | 0.8 | 3.3 |
| Madagascar | MDG | 2021 | 39.1 | 13.4 | 7.3 | 1.6 | 1.9 | 0.5 | 3.0 | 0.9 |

| | | | | | | | | | | |
|---|---|---|---|---|---|---|---|---|---|---|
| Malawi | MWI | 2015 | 34.8 | 10.4 | 3.1 | 0.7 | 4.3 | 1.0 | 1.0 | 1.8 |
| Mozambique | MOZ | 2011 | 39.7 | 17.5 | 5.3 | 1.8 | 7.9 | 2.1 | 1.4 | 4.5 |
| Namibia | NAM | 2013 | 22.9 | 7.7 | 8.2 | 2.7 | 4.5 | 1.1 | 1.8 | 1.8 |
| Rwanda | RWA | 2019 | 34.1 | 9.2 | 1.1 | 0.2 | 5.7 | 0.7 | 0.4 | 2.0 |
| South Africa | ZAF | 2016 | 24.3 | 8.0 | 2.7 | 0.9 | 13.1 | 3.7 | 0.4 | 3.7 |
| Tanzania | TZA | 2015 | 33.8 | 11.2 | 4.9 | 1.3 | 3.6 | 0.9 | 1.6 | 1.7 |
| Uganda | UGA | 2016 | 27.1 | 8.3 | 3.7 | 1.3 | 4.0 | 1.0 | 1.1 | 1.2 |
| Zambia | ZMB | 2018 | 34.7 | 11.6 | 4.0 | 1.3 | 5.1 | 1.3 | 1.0 | 2.5 |
| Zimbabwe | ZWE | 2015 | 25.4 | 7.4 | 3.5 | 1.2 | 6.3 | 1.7 | 0.6 | 2.3 |
| **Latin America and Caribbean** | | | **25.3** | **7.8** | **1.5** | **0.4** | **6.0** | **1.4** | **0.4** | **1.4** |
| Bolivia | BOL | 2003 | 33.5 | 11.3 | 1.9 | 0.8 | 9.3 | 2.1 | 0.5 | 3.8 |
| Brazil | BRA | 1996 | 15.4 | 4.2 | 2.8 | 1.0 | 6.3 | 1.4 | 0.4 | 1.1 |
| Colombia | COL | 2009 | 14.6 | 3.0 | 1.1 | 0.2 | 4.6 | 0.9 | 0.3 | 0.5 |
| Dominican Republic | DOM | 2013 | 7.8 | 2.0 | 2.5 | 0.7 | 7.4 | 2.0 | 0.4 | 1.0 |
| Guatemala | GTM | 2014 | 46.3 | 17.0 | 0.7 | 0.1 | 5.2 | 1.0 | 0.4 | 1.6 |
| Haiti | HTI | 2016 | 21.9 | 6.9 | 3.6 | 0.8 | 3.5 | 0.9 | 1.0 | 1.2 |
| Honduras | HND | 2011 | 25.9 | 7.5 | 1.4 | 0.3 | 4.9 | 1.1 | 0.5 | 0.8 |
| Nicaragua | NIC | 2001 | 26.2 | 8.8 | 2.3 | 0.9 | 7.7 | 2.5 | 0.7 | 2.2 |
| Paraguay | PRY | 1990 | 19.5 | 5.2 | 0.6 | 0.2 | 6.3 | 1.0 | 0.2 | 1.5 |
| Peru | PER | 2012 | 20.8 | 4.3 | 0.7 | 0.1 | 6.1 | 1.3 | 0.2 | 0.5 |
| **Middle East and North Africa** | | | **18.1** | **7.6** | **8.7** | **4.2** | **11.3** | **3.9** | **0.7** | **5.2** |
| Egypt | EGY | 2014 | 20.0 | 9.1 | 10.8 | 5.4 | 13.0 | 4.6 | 0.7 | 6.8 |
| Jordan | JOR | 2012 | 8.9 | 2.1 | 2.2 | 0.7 | 5.3 | 1.3 | 0.2 | 1.0 |
| Morocco | MAR | 2003 | 23.4 | 9.9 | 10.7 | 5.0 | 13.8 | 4.9 | 1.0 | 5.8 |
| **Eastern Europe and Central Asia** | | | **17.1** | **5.9** | **4.7** | **1.8** | **9.6** | **3.0** | **0.6** | **3.6** |
| Albania | ALB | 2017 | 12.4 | 3.7 | 2.1 | 0.7 | 16.4 | 4.6 | 0.2 | 4.3 |
| Armenia | ARM | 2015 | 10.3 | 3.4 | 4.7 | 1.6 | 13.3 | 4.3 | 0.2 | 3.4 |
| Azerbaijan | AZE | 2006 | 27.2 | 12.2 | 6.2 | 2.0 | 12.5 | 5.0 | 1.1 | 8.5 |
| Kazakhstan | KAZ | 1999 | 15.6 | 3.9 | 3.2 | 1.2 | 5.6 | 1.4 | 0.0 | 1.6 |
| Kyrgyzstan | KGZ | 2012 | 18.3 | 5.6 | 2.9 | 1.2 | 9.6 | 2.3 | 0.4 | 3.7 |
| Moldova | MDA | 2005 | 11.4 | 2.9 | 5.4 | 2.0 | 9.4 | 2.7 | 0.3 | 2.6 |
| Tajikistan | TJK | 2017 | 18.2 | 5.4 | 6.5 | 2.2 | 3.8 | 1.2 | 0.8 | 1.7 |
| Turkey | TUR | 2018 | 7.7 | 1.9 | 1.8 | 0.5 | 8.4 | 1.9 | 0.2 | 1.1 |
| Uzbekistan | UZB | 1996 | 34.6 | 19.3 | 11.4 | 6.1 | 18.2 | 8.8 | 1.8 | 10.1 |
| **East Asia and Pacific** | | | **36.7** | **15.0** | **15.3** | **5.7** | **3.8** | **1.6** | **4.6** | **2.3** |
| Cambodia | KHM | 2021 | 23.5 | 6.2 | 10.1 | 2.6 | 4.2 | 1.6 | 1.9 | 2.0 |
| Myanmar | MMR | 2015 | 30.7 | 8.7 | 6.2 | 1.3 | 1.6 | 0.3 | 1.6 | 0.6 |
| Timor-Leste | TLS | 2016 | 46.6 | 23.1 | 24.2 | 10.1 | 4.9 | 2.3 | 8.0 | 3.4 |
| **Global** | | | **31.3** | **12.4** | **10.3** | **3.8** | **4.6** | **1.4** | **2.8** | **2.1** |

**Appendix 6: Frequency of children exposed to any negative income shocks at different exposure windows by region and country**

| Country | Country code | Number of children 0-4 years | Income shock in the year of the interview | | Income shock in the year of birth | | Number of children 2-4 years | Income shock in all years of the first 1.000 days | |
|---|---|---|---|---|---|---|---|---|---|
| | | | n | % | n | % | | n | % |
| **South Asia** | | **566,341** | **195,805** | **34.6** | **305,700** | **54.0** | **337,713** | **114,281** | **33.8** |
| Bangladesh | BGD | 35,063 | 12,745 | 36.3 | 24,633 | 70.3 | 19,154 | 6,828 | 35.6 |
| India | IND | 495,218 | 151,669 | 30.6 | 250,637 | 50.6 | 298,000 | 95,832 | 32.2 |
| Maldives | MDV | 4,620 | 2,445 | 52.9 | 2,679 | 58.0 | 2,793 | 134 | 4.8 |
| Nepal | NPL | 20,359 | 18,950 | 93.1 | 18,169 | 89.2 | 11,061 | 6,951 | 62.8 |
| Pakistan | PAK | 11,081 | 9,996 | 90.2 | 9,582 | 86.5 | 6,705 | 4,536 | 67.7 |
| **West and Central Africa** | | **368,098** | **163,771** | **44.5** | **208,654** | **56.7** | **204,141** | **41,265** | **20.2** |
| Benin | BEN | 39,910 | 20,079 | 50.3 | 23,431 | 58.7 | 22,326 | 2,773 | 12.4 |
| Burkina Faso | BFA | 26,677 | 4,440 | 16.6 | 10,722 | 40.2 | 14,573 | 2 | 0.0 |
| Cameroon | CMR | 16,379 | 7,421 | 45.3 | 6,684 | 40.8 | 8,715 | 286 | 3.3 |
| Central African Republic | CAF | 2,351 | 2,023 | 86.0 | 1,550 | 65.9 | 740 | 0 | 0.0 |
| Chad | TCD | 19,885 | 606 | 3.0 | 8,269 | 41.6 | 11,729 | 1,382 | 11.8 |
| Congo | COG | 7,991 | 7,317 | 91.6 | 5,618 | 70.3 | 4,456 | 19 | 0.4 |
| Côte d'Ivoire | CIV | 8,522 | 4,731 | 55.5 | 4,391 | 51.5 | 3,749 | 216 | 5.8 |
| Democratic Republic of the Congo | COD | 11,169 | 9,267 | 83.0 | 10,510 | 94.1 | 6,465 | 4,572 | 70.7 |
| Gabon | GAB | 11,622 | 5,622 | 48.4 | 5,685 | 48.9 | 6,439 | 0 | 0.0 |
| Gambia | GMB | 6,710 | 5,469 | 81.5 | 3,193 | 47.6 | 3,728 | 39 | 1.0 |
| Ghana | GHA | 12,710 | 12,710 | 100.0 | 12,710 | 100.0 | 6,784 | 6,784 | 100.0 |
| Guinea | GIN | 13,200 | 6,047 | 45.8 | 7,329 | 55.5 | 7,635 | 1,707 | 22.4 |
| Liberia | LBR | 9,783 | 5,042 | 51.5 | 4,127 | 42.2 | 5,469 | 0 | 0.0 |
| Mali | MLI | 37,471 | 11,342 | 30.3 | 15,995 | 42.7 | 20,111 | 744 | 3.7 |
| Mauritania | MRT | 9,475 | 9,475 | 100.0 | 4,313 | 45.5 | 5,594 | 0 | 0.0 |
| Niger | NER | 12,347 | 1,782 | 14.4 | 7,413 | 60.0 | 6,096 | 940 | 15.4 |
| Nigeria | NGA | 64,515 | 27,066 | 42.0 | 53,062 | 82.2 | 37,240 | 18,964 | 50.9 |
| São Tomé and Príncipe | STP | 1,464 | 1,464 | 100.0 | 1,128 | 77.0 | 885 | 20 | 2.3 |
| Senegal | SEN | 39,481 | 12,636 | 32.0 | 13,348 | 33.8 | 22,809 | 1,209 | 5.3 |
| Sierra Leone | SLE | 10,114 | 6,044 | 59.8 | 7,299 | 72.2 | 5,773 | 1,608 | 27.9 |
| Togo | TGO | 6,322 | 3,188 | 50.4 | 1,877 | 29.7 | 2,825 | 0 | 0.0 |
| **Eastern and Southern Africa** | | **302,559** | **217,863** | **72.0** | **227,013** | **75.0** | **166,687** | **78,706** | **47.2** |
| Angola | AGO | 6,251 | 4,955 | 79.3 | 5,363 | 85.8 | 3,536 | 2,804 | 79.3 |
| Burundi | BDI | 9,405 | 9,405 | 100.0 | 8,755 | 93.1 | 5,436 | 4,283 | 78.8 |
| Comoros | COM | 3,321 | 1,176 | 35.4 | 1,027 | 30.9 | 1,641 | 119 | 7.3 |
| Eswatini | SWZ | 1,985 | 1,554 | 78.3 | 1,134 | 57.1 | 1,089 | 54 | 5.0 |
| Ethiopia | ETH | 35,547 | 25,084 | 70.6 | 26,046 | 73.3 | 21,624 | 13,474 | 62.3 |
| Kenya | KEN | 41,291 | 30,885 | 74.8 | 30,837 | 74.7 | 20,840 | 4,947 | 23.7 |
| Lesotho | LSO | 4,136 | 4,030 | 97.4 | 2,975 | 71.9 | 2,267 | 226 | 10.0 |

| | | | | | | | | | |
|---|---|---|---|---|---|---|---|---|---|
| Madagascar | MDG | 13,116 | 8,931 | 68.1 | 11,214 | 85.5 | 6,696 | 5,060 | 75.6 |
| Malawi | MWI | 29,794 | 29,794 | 100.0 | 28,778 | 96.6 | 16,684 | 14,027 | 84.1 |
| Mozambique | MOZ | 20,474 | 671 | 3.3 | 10,149 | 49.6 | 10,483 | 1,092 | 10.4 |
| Namibia | NAM | 10,685 | 8,018 | 75.0 | 6,915 | 64.7 | 5,458 | 1,607 | 29.4 |
| Rwanda | RWA | 25,145 | 14,047 | 55.9 | 15,478 | 61.6 | 14,653 | 3,608 | 24.6 |
| South Africa | ZAF | 1,032 | 1,032 | 100.0 | 1,032 | 100.0 | 628 | 627 | 99.8 |
| Tanzania | TZA | 29,452 | 28,801 | 97.8 | 26,239 | 89.1 | 16,457 | 9,973 | 60.6 |
| Uganda | UGA | 18,143 | 6,822 | 37.6 | 11,456 | 63.1 | 9,556 | 2,779 | 29.1 |
| Zambia | ZMB | 35,431 | 32,457 | 91.6 | 30,797 | 86.9 | 20,384 | 12,814 | 62.9 |
| Zimbabwe | ZWE | 17,351 | 10,201 | 58.8 | 8,818 | 50.8 | 9,255 | 1,212 | 13.1 |
| **Latin America and Caribbean** | | **228,793** | **136,295** | **59.6** | **156,399** | **68.4** | **132,397** | **65,659** | **49.6** |
| Bolivia | BOL | 18,116 | 14,817 | 81.8 | 17,105 | 94.4 | 10,278 | 8,703 | 84.7 |
| Brazil | BRA | 4,064 | 3,665 | 90.2 | 3,665 | 90.2 | 2,380 | 2,144 | 90.1 |
| Colombia | COL | 31,045 | 10,658 | 34.3 | 19,640 | 63.3 | 16,290 | 5,409 | 33.2 |
| Dominican Republic | DOM | 24,567 | 9,279 | 37.8 | 13,397 | 54.5 | 14,560 | 1,360 | 9.3 |
| Guatemala | GTM | 23,986 | 18,510 | 77.2 | 18,372 | 76.6 | 14,144 | 8,005 | 56.6 |
| Haiti | HTI | 19,331 | 18,958 | 98.1 | 17,773 | 91.9 | 11,047 | 8,660 | 78.4 |
| Honduras | HND | 18,602 | 9,055 | 48.7 | 14,313 | 76.9 | 11,091 | 6,269 | 56.5 |
| Nicaragua | NIC | 12,598 | 12,020 | 95.4 | 12,508 | 99.3 | 7,618 | 7,511 | 98.6 |
| Paraguay | PRY | 3,622 | 3,622 | 100.0 | 3,622 | 100.0 | 2,113 | 2,113 | 100.0 |
| Peru | PER | 72,862 | 35,711 | 49.0 | 36,004 | 49.4 | 42,876 | 15,485 | 36.1 |
| **Middle East and North Africa** | | **97,254** | **70,612** | **72.6** | **72,420** | **74.5** | **58,347** | **31,823** | **54.5** |
| Egypt | EGY | 60,172 | 50,189 | 83.4 | 51,702 | 85.9 | 35,907 | 24,660 | 68.7 |
| Jordan | JOR | 27,249 | 15,881 | 58.3 | 17,385 | 63.8 | 16,475 | 7,163 | 43.5 |
| Morocco | MAR | 9,833 | 4,542 | 46.2 | 3,333 | 33.9 | 5,965 | 0 | 0.0 |
| **Eastern Europe and Central Asia** | | **46,553** | **30,867** | **66.3** | **31,123** | **66.9** | **27,214** | **11,728** | **43.1** |
| Albania | ALB | 3,711 | 822 | 22.2 | 918 | 24.7 | 2,368 | 8 | 0.3 |
| Armenia | ARM | 5,557 | 2,214 | 39.8 | 2,049 | 36.9 | 3,322 | 246 | 7.4 |
| Azerbaijan | AZE | 1,901 | 597 | 31.4 | 342 | 18.0 | 1,126 | 0 | 0.0 |
| Kazakhstan | KAZ | 1,285 | 1,285 | 100.0 | 1,285 | 100.0 | 576 | 576 | 100.0 |
| Kyrgyzstan | KGZ | 4,809 | 2,874 | 59.8 | 4,376 | 91.0 | 2,485 | 1,702 | 68.5 |
| Moldova | MDA | 1,259 | 506 | 40.2 | 470 | 37.3 | 721 | 0 | 0.0 |
| Tajikistan | TJK | 9,927 | 9,378 | 94.5 | 7,205 | 72.6 | 5,959 | 2,637 | 44.3 |
| Turkey | TUR | 17,128 | 12,215 | 71.3 | 13,502 | 78.8 | 10,334 | 6,236 | 60.3 |
| Uzbekistan | UZB | 976 | 976 | 100.0 | 976 | 100.0 | 323 | 323 | 100.0 |
| **East Asia and Pacific** | | **34,300** | **11,927** | **34.8** | **11,752** | **34.3** | **20,305** | **3,070** | **15.1** |
| Cambodia | KHM | 17,155 | 4,355 | 25.4 | 5,364 | 31.3 | 9,626 | 24 | 0.2 |
| Myanmar | MMR | 4,088 | 646 | 15.8 | 865 | 21.2 | 2,455 | 0 | 0.0 |
| Timor-Leste | TLS | 13,057 | 6,926 | 53.0 | 5,523 | 42.3 | 8,224 | 3,046 | 37.0 |
| **Global** | | **1,643,898** | **827,140** | **50.3** | **1,013,061** | **61.6** | **946,804** | **346,532** | **36.6** |

**Appendix 7: Full models of the adjusted association between exposure to income shocks in the year of the interview and child malnutrition outcomes**

| Year of the interview | Stunting (HAZ<-2) | Severe stunting (HAZ<-3) | Wasting (WHZ<-2) | Severe wasting (WHZ<-3) | Overweight (WHZ>2) | Obesity (WHZ>3) | WaSt (WHZ<-2 & HAZ<-2) | DBM (HAZ<-2 & WHZ>2) |
|---|---|---|---|---|---|---|---|---|
| | PR (95%CI) | PR (95%CI) | PR (95%CI) | PR (95%CI) | PR (95%CI) | PR (95%CI) | PR (95%CI) | PR (95%CI) |
| | p-value | p-value | p-value | p-value | p-value | p-value | p-value | p-value |
| Any negative income shock | 0.995 (0.986-1.005) | 1.020 (1.003-1.037) | 1.054 (1.029-1.080) | 1.127 (1.079-1.176) | 1.036 (1.007-1.067) | 1.093 (1.029-1.161) | 1.027 (0.987-1.070) | 1.046 (0.997-1.097) |
| | 0.334 | 0.021 | <0.001 | <0.001 | 0.016 | 0.004 | 0.189 | 0.067 |
| Rural place of residence | 1.077 (1.065-1.089) | 1.097 (1.076-1.119) | 0.955 (0.931-0.980) | 0.933 (0.891-0.978) | 1.004 (0.974-1.034) | 1.025 (0.967-1.087) | 0.979 (0.933-1.027) | 1.035 (0.986-1.086) |
| | <0.001 | <0.001 | 0.001 | 0.004 | 0.805 | 0.405 | 0.385 | 0.167 |
| Unimproved toilet facility | 1.029 (1.020-1.038) | 1.057 (1.042-1.073) | 1.063 (1.040-1.086) | 1.068 (1.027-1.110) | 0.966 (0.938-0.994) | 0.992 (0.938-1.048) | 1.100 (1.060-1.142) | 0.982 (0.941-1.025) |
| | <0.001 | <0.001 | <0.001 | 0.001 | 0.019 | 0.764 | <0.001 | 0.410 |
| Wealth index quintile 1 | 1.602 (1.576-1.628) | 1.866 (1.812-1.921) | 1.340 (1.291-1.391) | 1.410 (1.318-1.510) | 0.815 (0.780-0.852) | 0.831 (0.761-0.907) | 1.917 (1.788-2.056) | 1.158 (1.077-1.246) |
| | <0.001 | <0.001 | <0.001 | <0.001 | <0.001 | <0.001 | <0.001 | <0.001 |
| Wealth index quintile 2 | 1.511 (1.487-1.535) | 1.692 (1.644-1.741) | 1.242 (1.197-1.289) | 1.255 (1.174-1.342) | 0.810 (0.777-0.844) | 0.750 (0.691-0.814) | 1.697 (1.584-1.819) | 1.060 (0.990-1.135) |
| | <0.001 | <0.001 | <0.001 | <0.001 | <0.001 | <0.001 | <0.001 | 0.096 |
| Wealth index quintile 3 | 1.416 (1.395-1.439) | 1.534 (1.492-1.578) | 1.167 (1.126-1.209) | 1.199 (1.123-1.280) | 0.846 (0.814-0.879) | 0.850 (0.788-0.917) | 1.500 (1.402-1.605) | 1.084 (1.015-1.157) |
| | <0.001 | <0.001 | <0.001 | <0.001 | <0.001 | <0.001 | <0.001 | 0.016 |
| Wealth index quintile 4 | 1.293 (1.274-1.313) | 1.361 (1.325-1.398) | 1.118 (1.082-1.156) | 1.125 (1.059-1.195) | 0.870 (0.841-0.901) | 0.835 (0.779-0.895) | 1.340 (1.256-1.431) | 1.034 (0.973-1.100) |
| | <0.001 | <0.001 | <0.001 | <0.001 | <0.001 | <0.001 | <0.001 | 0.284 |
| Maternal age at birth in years | 0.992 (0.991-0.993) | 0.991 (0.990-0.992) | 1.002 (1.000-1.004) | 1.002 (0.999-1.005) | 1.005 (1.003-1.007) | 1.007 (1.004-1.011) | 0.998 (0.996-1.001) | 0.994 (0.991-0.997) |
| | <0.001 | <0.001 | 0.011 | 0.157 | <0.001 | <0.001 | 0.249 | <0.001 |
| Maternal education <= 5 years | 1.208 (1.196-1.219) | 1.366 (1.343-1.389) | 1.173 (1.145-1.201) | 1.228 (1.177-1.281) | 0.929 (0.904-0.955) | 0.944 (0.896-0.996) | 1.465 (1.402-1.531) | 1.103 (1.058-1.151) |
| | <0.001 | <0.001 | <0.001 | <0.001 | <0.001 | 0.035 | <0.001 | <0.001 |
| Maternal parity >= 3 births | 1.084 (1.074-1.094) | 1.104 (1.086-1.121) | 1.022 (0.998-1.045) | 1.024 (0.983-1.067) | 0.908 (0.883-0.934) | 0.897 (0.849-0.948) | 1.077 (1.036-1.121) | 1.006 (0.963-1.051) |
| | <0.001 | <0.001 | 0.067 | 0.251 | <0.001 | <0.001 | <0.001 | 0.784 |
| Female child | 0.885 (0.879-0.891) | 0.828 (0.819-0.838) | 0.855 (0.841-0.870) | 0.793 (0.769-0.818) | 0.891 (0.872-0.911) | 0.895 (0.858-0.934) | 0.720 (0.699-0.742) | 0.885 (0.856-0.914) |
| | <0.001 | <0.001 | <0.001 | <0.001 | <0.001 | <0.001 | <0.001 | <0.001 |
| Child's age in months | 1.010 (1.010-1.010) | 1.011 (1.010-1.011) | 0.982 (0.981-0.982) | 0.975 (0.974-0.976) | 0.986 (0.985-0.986) | 0.981 (0.979-0.982) | 0.995 (0.994-0.996) | 0.990 (0.989-0.991) |
| | <0.001 | <0.001 | <0.001 | <0.001 | <0.001 | <0.001 | <0.001 | <0.001 |
| Child born at medical facility | 0.876 (0.869-0.883) | 0.800 (0.789-0.812) | 0.872 (0.854-0.891) | 0.838 (0.807-0.870) | 1.073 (1.045-1.101) | 1.062 (1.010-1.117) | 0.768 (0.740-0.798) | 0.960 (0.924-0.998) |
| | <0.001 | <0.001 | <0.001 | <0.001 | <0.001 | 0.019 | <0.001 | 0.042 |

Robust Poisson regression models adjusted for country and time (years) fixed effects and the following confounding variables: rural place of residence (ref. urban), unimproved toilet facility (ref. improved), wealth index quintile (ref. 5 richest), maternal age at birth (year), maternal education <= 5 years (ref. >5 years), maternal parity >= 3 births (ref. <3 births), female child (ref. male), child age (month), child born at medical facility (ref. others). Any negative income shock (ref. no negative income shock, i.e. income growth equal to or greater than zero). HAZ: height-for-age z-score, WHZ: weight-for-height z-score, WaSt: concurrent wasting and stunting, DBM: double burden of malnutrition, PR: prevalence ratio, 95% CI: 95% confidence interval. Analysis included children under five years of age (n=1,643,898).

**Appendix 8: Models with interaction term between income shock and wealth quintiles for each malnutrition outcome**

| | **Stunting** (HAZ<-2) | **Severe stunting** (HAZ<-3) | **Wasting** (WHZ<-2) | **Severe wasting** (WHZ<-3) | **Overweight** (WHZ>2) | **Obesity** (WHZ>3) | **WaSt** (WHZ<-2 & HAZ<-2) | **DBM** (HAZ<-2 & WHZ>2) |
|---|---|---|---|---|---|---|---|---|
| | PR (95%CI) p-value | PR (95%CI) p-value | PR (95%CI) p-value | PR (95%CI) p-value | PR (95%CI) p-value | PR (95%CI) p-value | PR (95%CI) p-value | PR (95%CI) p-value |
| **Year of interview** | | | | | | | | |
| Income shock# Wealth quintile 1 | 0.996 (0.981-1.010) 0.562 | 1.014 (0.988-1.041) 0.299 | 1.198 (1.164-1.232) <0.001 | 1.164 (1.108-1.222) <0.001 | 1.024 (0.981-1.068) 0.281 | 1.055 (0.976-1.140) 0.178 | 1.197 (1.129-1.270) <0.001 | 1.093 (1.027-1.165) 0.006 |
| Income shock# Wealth quintile 2 | 1.015 (1.000-1.030) 0.057 | 1.042 (1.014-1.071) 0.003 | 1.077 (1.046-1.109) <0.001 | 1.063 (1.011-1.118) 0.018 | 1.090 (1.044-1.137) <0.001 | 1.153 (1.066-1.248) <0.001 | 1.127 (1.060-1.197) <0.001 | 1.165 (1.092-1.242) <0.001 |
| Income shock# Wealth quintile 3 | 1.050 (1.034-1.066) <0.001 | 1.118 (1.087-1.150) <0.001 | 1.110 (1.078-1.144) <0.001 | 1.119 (1.063-1.178) <0.001 | 1.065 (1.021-1.111) 0.003 | 1.139 (1.054-1.230) 0.001 | 1.153 (1.083-1.227) <0.001 | 1.199 (1.124-1.280 <0.001 |
| Income shock# Wealth quintile 4 | 1.045 (1.028-1.062) <0.001 | 1.088 (1.057-1.121) <0.001 | 1.089 (1.056-1.123) <0.001 | 1.077 (1.022-1.135) 0.005 | 1.000 (0.960-1.042) 0.986 | 1.045 (0.968-1.127) 0.259 | 1.148 (1.075-1.226) <0.001 | 1.111 (1.041-1.186) 0.002 |
| Income shock# Wealth quintile 5 | Ref. | Ref. | Ref. | Ref. | Ref. | Ref. | Ref. | Ref. |
| **Statistical tests for interaction** | | | | | | | | |
| P-value for Wald test | <0.0001 | <0.0001 | <0.0001 | <0.0001 | 0.0001 | 0.0011 | <0.0001 | <0.0001 |
| P-value for likelihood-ratio test | <0.0001 | <0.0001 | <0.0001 | <0.0001 | <0.0001 | <0.0001 | <0.0001 | <0.0001 |

Robust Poisson regression models. Any negative income shock (ref. no negative income shock, i.e. income growth equal to or greater than zero). HAZ: height-for-age z-score, WHZ: weight-for-height z-score, WaSt: concurrent wasting and stunting, DBM: double burden of malnutrition, PR: prevalence ratio, 95% CI: 95% confidence interval. Analysis included children under five years of age (n=1,643,898).

**Appendix 9: Adjusted models with HAZ and WHZ as dependent continuous variables**

| Year of the interview† | HAZ (height-for-age z-score) | | WHZ (weight-for-height z-score) | |
|---|---|---|---|---|
| | Coef. (95% CI) | p-value | Coef. (95% CI) | p-value |
| Any negative income shock | 0.018 (0.007, 0.028) | 0.001 | -0.012 (-0.021, -0.002) | 0.013 |
| Rural place of residence | -0.068 (-0.079, -0.057) | <0.001 | 0.029 (0.019, 0.039) | <0.001 |
| Unimproved toilet facility | -0.039 (-0.050, -0.029) | <0.001 | -0.042 (-0.050, -0.033) | <0.001 |
| Wealth index quintile 1 (poorest) | -0.524 (-0.541, -0.508) | <0.001 | -0.180 (-0.194, -0.166) | <0.001 |
| Wealth index quintile 2 | -0.443 (-0.458, -0.428) | <0.001 | -0.142 (-0.156, -0.129) | <0.001 |
| Wealth index quintile 3 | -0.356 (-0.370, -0.341) | <0.001 | -0.101 (-0.114, -0.088) | <0.001 |
| Wealth index quintile 4 | -0.255 (-0.268, -0.242) | <0.001 | -0.075 (-0.086, -0.063) | <0.001 |
| Maternal age at birth in years | 0.012 (0.011, 0.012) | <0.001 | -0.001 (-0.001, 0.000) | 0.013 |
| Maternal education <= 5 years | -0.210 (-0.220, -0.200) | <0.001 | -0.078 (-0.086, -0.069) | <0.001 |
| Maternal parity >= 3 births | -0.085 (-0.096, -0.075) | <0.001 | -0.013 (-0.022, -0.004) | 0.004 |
| Female child | 0.134 (0.126, 0.142) | <0.001 | 0.018 (0.011, 0.025) | <0.001 |
| Child's age in months | -0.019 (-0.020, -0.019) | <0.001 | 0.002 (0.002, 0.002) | <0.001 |
| Child born at medical facility | 0.166 (0.156, 0.175) | <0.001 | 0.094 (0.085, 0.102) | <0.001 |

| Year of birth† | HAZ (height-for-age z-score) | | WHZ (weight-for-height z-score) | |
|---|---|---|---|---|
| | Coef. (95% CI) | p-value | Coef. (95% CI) | p-value |
| Any negative income shock | -0.033 (-0.042, -0.023) | <0.001 | 0.006 (-0.002, 0.014) | 0.163 |
| Rural place of residence | -0.069 (-0.080, -0.058) | <0.001 | 0.029 (0.020, 0.039) | <0.001 |
| Unimproved toilet facility | -0.040 (-0.050, -0.030) | <0.001 | -0.041 (-0.050, -0.033) | <0.001 |
| Wealth index quintile 1 (poorest) | -0.527 (-0.543, -0.510) | <0.001 | -0.179 (-0.194, -0.165) | <0.001 |
| Wealth index quintile 2 | -0.444 (-0.459, -0.428) | <0.001 | -0.142 (-0.156, -0.129) | <0.001 |
| Wealth index quintile 3 | -0.356 (-0.370, -0.342) | <0.001 | -0.101 (-0.113, -0.088) | <0.001 |
| Wealth index quintile 4 | -0.256 (-0.269, -0.243) | <0.001 | -0.074 (-0.086, -0.063) | <0.001 |
| Maternal age at birth in years | 0.012 (0.011, 0.012) | <0.001 | -0.001 (-0.001, 0.000) | 0.014 |
| Maternal education <= 5 years | -0.210 (-0.220, -0.200) | <0.001 | -0.078 (-0.086, -0.069) | <0.001 |
| Maternal parity >= 3 births | -0.085 (-0.096, -0.075) | <0.001 | -0.013 (-0.022, -0.004) | 0.004 |
| Female child | 0.134 (0.126, 0.142) | <0.001 | 0.018 (0.011, 0.025) | <0.001 |
| Child's age in months | -0.019 (-0.020, -0.019) | <0.001 | 0.002 (0.002, 0.002) | <0.001 |
| Child born at medical facility | 0.165 (0.155, 0.175) | <0.001 | 0.094 (0.086, 0.102) | <0.001 |

| All years of the first 1.000 days†† | HAZ (height-for-age z-score) | | WHZ (weight-for-height z-score) | |
|---|---|---|---|---|
| | Coef. (95% CI) | p-value | Coef. (95% CI) | p-value |
| Any negative income shock | -0.005 (-0.018, 0.007) | 0.407 | 0.032 (0.021, 0.043) | <0.001 |
| Rural place of residence | -0.083 (-0.096, -0.069) | <0.001 | 0.034 (0.022, 0.045) | <0.001 |
| Unimproved toilet facility | -0.037 (-0.049, -0.025) | <0.001 | -0.035 (-0.046, -0.025) | <0.001 |
| Wealth index quintile 1 (poorest) | -0.598 (-0.617, -0.578) | <0.001 | -0.131 (-0.148, -0.113) | <0.001 |
| Wealth index quintile 2 | -0.511 (-0.529, -0.493) | <0.001 | -0.100 (-0.116, -0.083) | <0.001 |
| Wealth index quintile 3 | -0.410 (-0.427, -0.393) | <0.001 | -0.067 (-0.082, -0.051) | <0.001 |
| Wealth index quintile 4 | -0.293 (-0.308, -0.277) | <0.001 | -0.058 (-0.072, -0.044) | <0.001 |
| Maternal age at birth in years | 0.014 (0.014, 0.015) | <0.001 | 0.001 (0.000, 0.001) | 0.150 |
| Maternal education <= 5 years | -0.240 (-0.252, -0.229) | <0.001 | -0.048 (-0.058, -0.038) | <0.001 |
| Maternal parity >= 3 births | -0.139 (-0.152, -0.127) | <0.001 | 0.005 (-0.006, 0.016) | 0.390 |
| Female child | 0.065 (0.056, 0.075) | <0.001 | -0.028 (-0.037, -0.020) | <0.001 |
| Child's age in months | 0.004 (0.004, 0.005) | <0.001 | -0.002 (-0.003, -0.002) | <0.001 |
| Child born at medical facility | 0.194 (0.182, 0.205) | <0.001 | 0.068 (0.058, 0.078) | <0.001 |

Linear regression models adjusted for country and year fixed effects and the following confounding variables: rural place of residence (vs. urban), unimproved toilet facility (vs. improved), wealth index quintile (vs. 5 richest), maternal age at birth (year), maternal education <= 5 years (vs. >5 years), maternal parity >= 3 births (vs. <3 births), female child (vs. male), child's age (month), child born at medical facility (vs. others). Any negative income shock (ref. no negative income shock, i.e. income growth equal to or greater than zero). 95% CI: 95% confidence interval. † All children under five years of age (n=1,643,898). †† Only children aged two years or older (n=946,804).

**Appendix 10: Consistency check between WID's pretax income and World Bank's national accounts**

This figure presents the annual growth rate (y-axis) over the period from 1980 to 2022 (x-axis) for national pretax income per capita from the WID (black line) and GNI per capita (green line) and GDP per capita (yellow line) from the World Bank. In general, the observed trends suggest consistency between income growth rates as reported by the WID and the national economic indicators of GNI and GDP from the World Bank.

WID: World Inequality Database, GNI: Gross National Income, GDP: Gross Domestic Product.

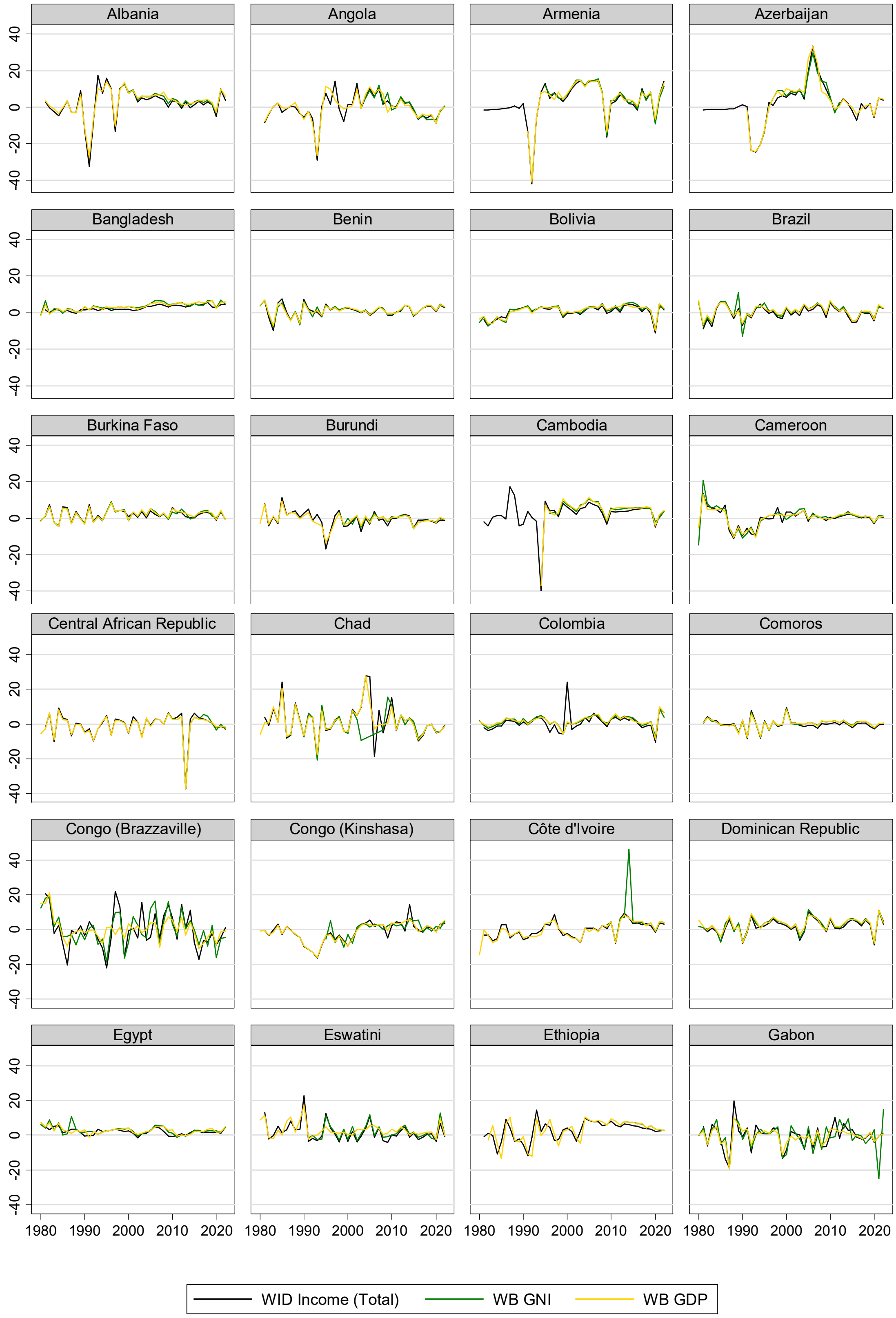

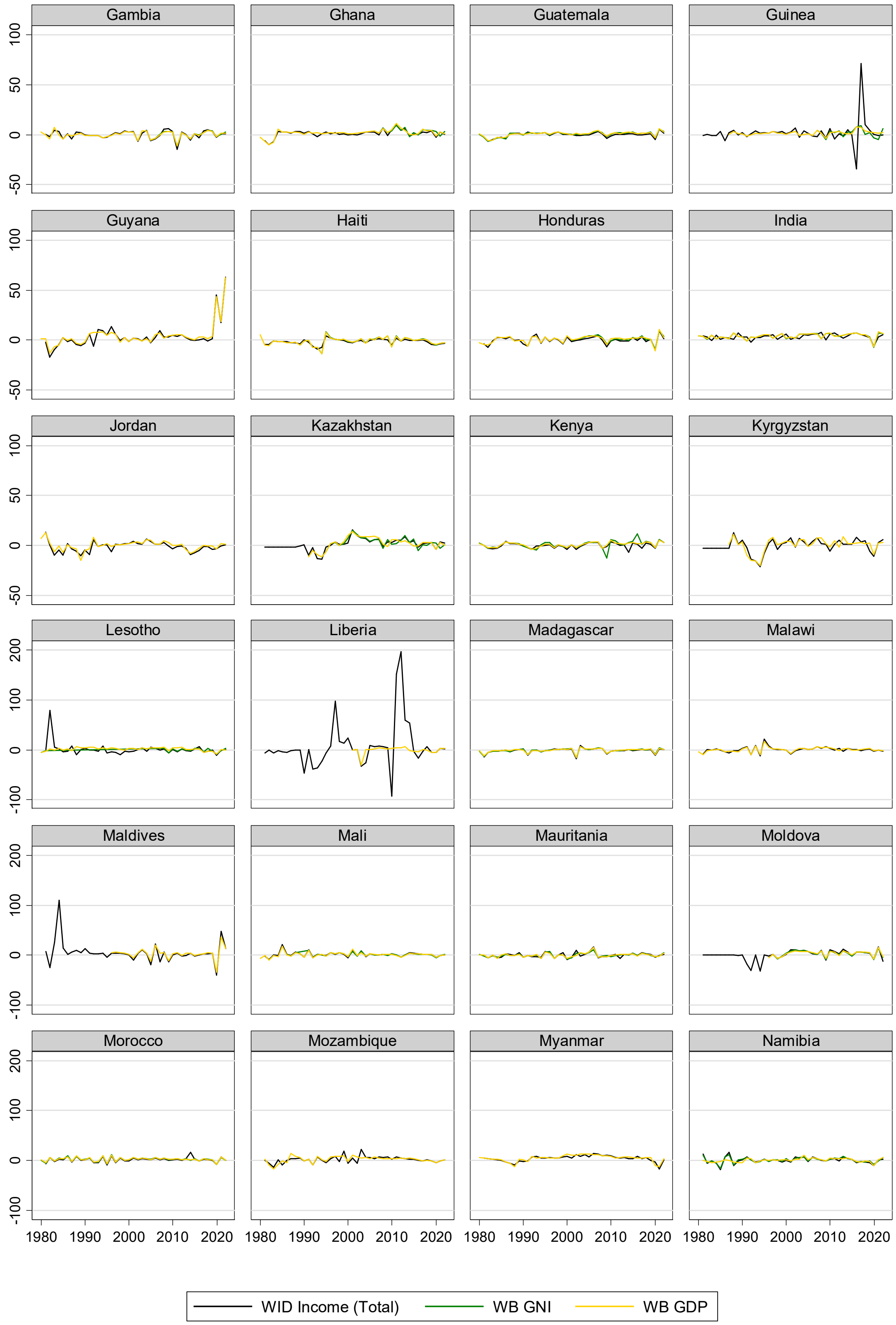

Gambia
Ghana
Guatemala
Guinea
Guyana
Haiti
Honduras
India
Jordan
Kazakhstan
Kenya
Kyrgyzstan
Lesotho
Liberia
Madagascar
Malawi
Maldives
Mali
Mauritania
Moldova
Morocco
Mozambique
Myanmar
Namibia
WID Income (Total)
WB GNI
WB GDP

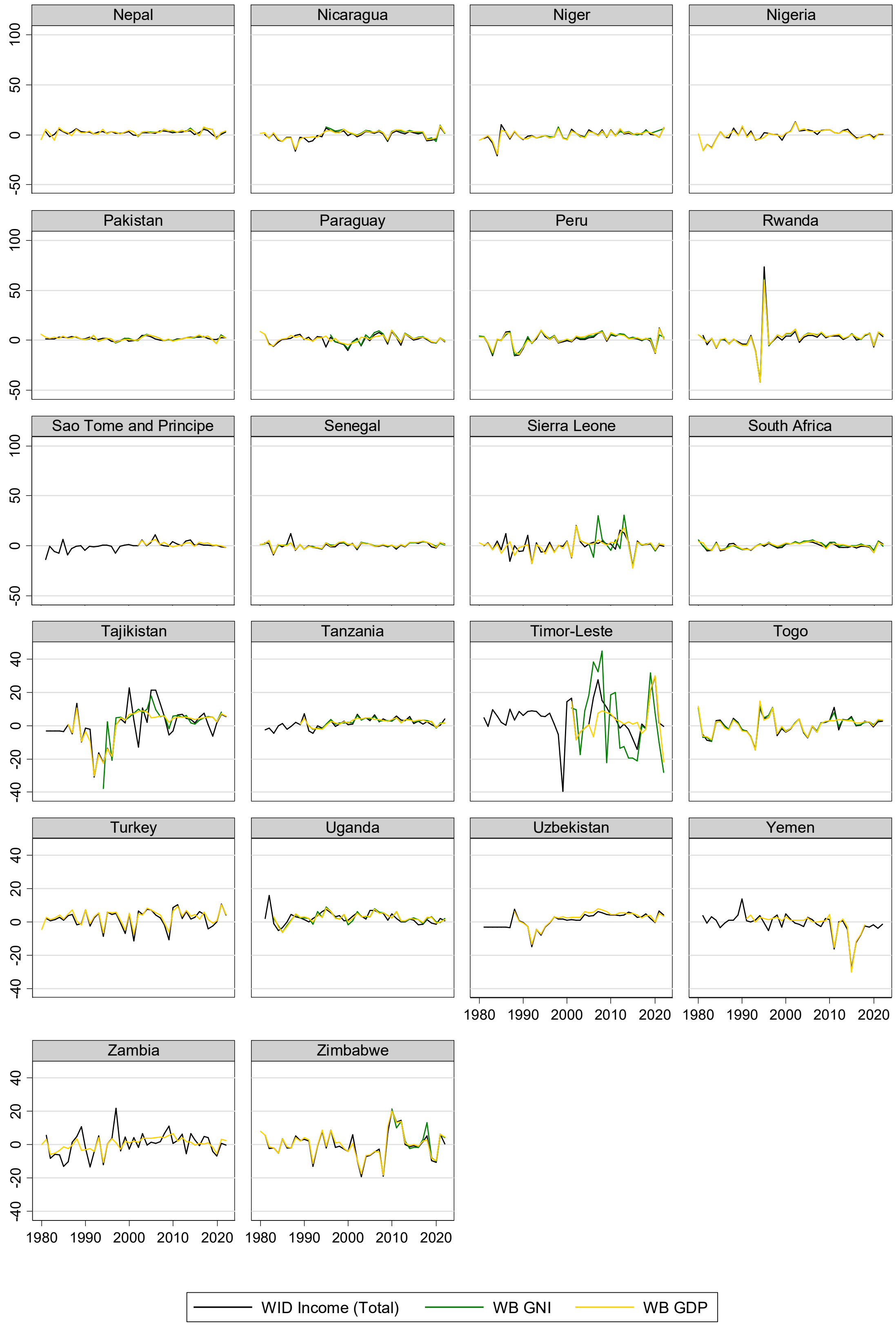
Nepal
Nicaragua
Niger
Nigeria
Pakistan
Paraguay
Peru
Rwanda
Sao Tome and Principe
Senegal
Sierra Leone
South Africa
Tajikistan
Tanzania
Timor-Leste
Togo
Turkey
Uganda
Uzbekistan
Yemen
Zambia
Zimbabwe
-50
0
50
100
-40
-20
0
20
40
1980
1990
2000
2010
2020
WID Income (Total)
WB GNI
WB GDP

**Appendix 11: Pilot analyses with multilevel mixed-effects Poisson and simple Poisson regression models**

The hierarchical structure of our study data, with individuals nested within surveys and surveys nested within countries, suggests that a multilevel mixed-effects approach would be ideal. However, implementing such models for the full dataset presented computational challenges, including numerical overflow and model convergence failures. We conducted a pilot analysis using a two-level mixed-effects Poisson model (unweighted and unadjusted) on a subset of 10 countries to explore the feasibility of a mixed-effects approach. The model successfully converged and yielded an incidence rate ratio (IRR) of 1.69 (95% CI: 1.12–2.56, p= 0.013). For comparison, a simple Poisson regression model with the same specifications produced a similar IRR of 1.67 (95% CI: 1.56–1.77, p<0.001), suggesting that the simpler model approximates the estimates of the hierarchical model in this case.
